# Scientists' Warning to Humanity: The Need to Begin Teaching Critical and Systems Thinking Early in Life

**DOI:** 10.1111/1751-7915.70270

**Published:** 2025-12-15

**Authors:** Kenneth Timmis, Fernando Baquero, Rup Lal, Lara Raquel Pinto Amorim, Pablo Ivan Nikel, Jasvinder Kaur, Utkarsh Sood, Pushp Lata, Shalini Singh, Jake M. Robinson, Max Chavarria, Willy Verstraete, Patricia Bernal, Horia Banciu, Karen Steward, Joachim Frey, Antoine Danchin, Anna Karnkowska, Oleg Kotsyurbenko, Cristina Silva Pereira, Eric S. Boyd, John E. Hallsworth, Olga Nunes, Zulema Udaondo, Wei Huang, Yun Wang, Zeynep Ceren Karahan, Pilar Junier, Eliora Ron, Juan Luis Ramos

**Affiliations:** ^1^ Institute for Microbiology Technical University of Braunschweig Braunschweig Germany; ^2^ Ramón y Cajal Institute for Health Research (IRYCIS), Department of Microbiology, Ramón y Cajal University Hospital Madrid Spain; ^3^ Acharya Narendra Dev College University of Delhi New Delhi India; ^4^ Department of Biology, Faculty of Sciences University of Porto and IB2Lab‐LAQV‐REQUIMTE—Associated Laboratory for Green Chemistry of the Network of Chemistry and Technology Porto Portugal; ^5^ Systems Environmental Microbiology Group, the Novo Nordisk Foundation Center for Biosustainability Technical University of Denmark Lyngby Denmark; ^6^ Department of Zoology Gargi College, University of Delhi New Delhi India; ^7^ Department of Zoology Kirori Mal College, University of Delhi Delhi India; ^8^ Department of Zoology University of Delhi Delhi India; ^9^ Department of Biotechnology Helmholtz Zentrum Dresden Rossendorf Dresden Germany; ^10^ College of Science and Engineering, Flinders University Bedford Park South Australia Australia; ^11^ Escuela de Química, CIPRONA, Universidad de Costa Rica & Centro Nacional de Innovaciones Biotecnológicas (CENIBiot) San José Costa Rica; ^12^ Center for Microbial Ecology and Technology (CMET) Ghent University Ghent Belgium; ^13^ Department of Microbiology Universidad de Sevilla Sevilla Spain; ^14^ Department of Molecular Biology and Biotechnology Babeș‐Bolyai University Cluj‐Napoca Romania; ^15^ Technology Networks Suffolk UK; ^16^ Vetsuisse Faculty, University of Bern Bern Switzerland; ^17^ School of Biomedical Sciences, Li Kashing Faculty of Medicine, the University of Hong Kong, SAR Hong Kong China; ^18^ Institute of Evolutionary Biology, Faculty of Biology, Biological and Chemical Research Centre, University of Warsaw Warsaw Poland; ^19^ Higher School of Ecology, Yugra State University Khanty‐Mansiysk Russia; ^20^ Instituto de Tecnologia Química e Biológica António Xavier Oeiras Portugal; ^21^ Department of Microbiology and Cell Biology Montana State University Bozeman Montana USA; ^22^ Institute for Global Food Security, Queen's University Belfast UK; ^23^ LEPABE—Laboratory for Process Engineering, Environment, Biotechnology and Energy, Faculty of Engineering University of Porto Porto Portugal; ^24^ Centro Nacional de Biotecnología, CSIC Madrid Spain; ^25^ Department of Engineering Science University of Oxford Oxford UK; ^26^ Oxford Suzhou Center for Advanced Research Suzhou China; ^27^ Department of Medical Microbiology Ankara University School of Medicine Ankara Turkey; ^28^ Laboratory of Microbiology University of Neuchâtel Neuchâtel Switzerland; ^29^ The Shmunis School of Biomedicine and Cancer Research Tel Aviv University Tel Aviv Israel; ^30^ Consejo Superior de Investigaciones Cientificas, Estación Experimental del Zaidín Granada Spain

## Abstract

We live in a time of global crises: a deteriorating environment that is struggling to provide all the resources and services we demand of it, changing climate and its consequences for the biosphere, its habitats, inhabitants and biodiversity, conflicts‐divisive ideologies‐competition for resources, increasing societal inequalities and human deprivations, and a youth mental health pandemic, to name but just a few. Most of these crises are self‐made, the result of human decisions, and their acceptance/toleration by society. Policies and practices at all levels of society that created, exacerbate and launch new crises are, at worst, self‐serving and, at best, faulted through a lack of understanding. In democracies, citizens can hold decision‐makers to account but, to do this, they must understand the issues and be able to imagine better policies. We also live in a digital world in which a flood of mostly inconsequential information and misinformation pollutes our brains, enhancing pre‐existing biases and creating new ones, and numbing our mental ability to think clearly and reach sensible decisions. But sensible decisions are urgently needed at all levels to fix problems and reduce future self‐harm. Sensible decisions require sourcing the best available relevant information, and a process to convert information into understanding, understanding into clear decision options, and the choice of a decision option that leads to an action that represents best practice. Critical thinking is the enabling cognitive process of this decision pathway, because it selects the best available information through demanding evidence‐basing, seeks critical discourse between experts and stakeholders that agnostically explores solution space to find plausible options, and whittles down options inter alia through plausibility, due diligence, bottleneck analysis, cost‐benefit analysis, and benchmarking filtering. Crucially, it rejects biases, influencing factors, and other constraints on options, and is an effective barrier to the information flood. The problem is that critical thinking capacity is not widely available among either decision makers or stakeholders. There is an urgent need to rapidly roll out effective education programmes in which critical thinking teaching is solidly embedded. Since biases accumulate with age, the teaching of critical thinking must begin with the very young. However, the very young are not able to comprehend the complex abstract issues underpinning critical thinking. Embedding the teaching of critical thinking in a suitable educational context, and integrating it into curricula, is another challenge. To address these two challenges, the International Microbiology Literacy Initiative is developing a storytelling programme for children, called the Critical Thinking MicroChats Gallery, within the curriculum of societally relevant microbiology it is creating. MicroChats illustrate the principal practical elements of critical thinking, like bottlenecks, cost: benefit, benchmarking, the need for discussions and other points of view, employing readily relatable, relevant microbially centric scenarios. MicroChats suggest class discussion topics to encourage children to imagine the application of each element in other contexts to reinforce principles and hone critical thinking skills. Critical thinking, and especially the cultivation of the habit of asking ‘why’ and requiring plausible justification for policies/actions, is a shield against bias, prejudice, propaganda, misinformation and the incessant pressures of social media. It promotes a healthy mind and the attainment of the developmental potential of individuals. Increasing critical thinking in society will raise the quality of decision making at all levels and thereby improve sustainability/reduce the human footprint on our planet, and promote the individual sense of responsibility and global citizenship necessary for the improvement of the condition of humanity and its relationship with Planet Earth.

## 1 The Quality of Thinking Determines the Quality of Decisions and Their Outcomes

### 1.1 Decisions guide or influence all facets of human life, including the creation and mitigation of global crises

From childhood onwards, people form opinions, solve problems, respond to others, and make judgements that have consequences. As individuals gain greater influence—whether as parents, educators, leaders or policymakers—the impact of their decisions multiplies. We are all stewards of lives to some degree or other: at a minimum, of our own lives and the impact of our lives on others, and at the other end of the spectrum, political and religious leaders, the heads of multinational organisations and private enterprises, and, money‐empowered individuals, may make decisions that have profound effects on humanity, the health of the planet and its biosphere. Even if we are not stewards at higher levels of decision making, we are still stakeholders in many aspects of it and can and should, where appropriate, influence such decisions and hold decision makers to account.

**Bad Decisions Generally Engender Bad Outcomes**

Decisions have consequences: this is their purpose. The quality of decisions largely determines the quality of the outcomes. The consequences of bad decisions can range from the relatively unimportant to the catastrophic; there is a litany of historical bad decisions that had disastrous consequences for those affected by them, though not necessarily for those who took them (https://historycollection.com/10‐terrible‐decisions‐made‐world‐leaders‐throughout‐history/). There is a huge need to minimise the frequency of bad decisions in the world by addressing the key elements that influence and determine their qualities and consequences, in particular the quality of thinking.

### 1.2 Decisions are usually the result of thinking

Although some decisions are automatic (instinct‐driven or prescribed by rules‐predetermined by custom, culture or computer algorithms), most are outcomes of deliberations, often by multiple people who discuss and exchange their ideas and opinions (Weigand 2008), all of which are arrived at through the process of thinking.

Thinking is the cognitive process by which we interpret, process, manipulate and evolve applications from information. It encompasses functions such as analysing and contextualising, inferring, reasoning, creativity, synthesis, evaluation, developing understanding, judging and developing concepts, imagination and abstract thought, and recognising what is not known and seeking required information—the search for truth—often through the formulation of pertinent questions. It draws on cognitive, emotional and sensory processes/inputs and forms the basis of both individual understanding and social communication (Davies and Barnett 2015). When grounded in truth, it enables us to comprehend our environment, respond and adapt to changes in it, and confront challenges. Thinking can lead to the transformation of primary rough and messy information into new knowledge, of knowledge into understanding, the navigation of complexity, the formation of opinions, the solution of problems, participation in discussions with others, the formation of judgements, dealing with challenges and, crucially, making choices and decisions.

Thinking is not only a private process but also a social one; it is the basis of communication with others. Exchanging our thoughts, opinions and conclusions with others is of crucial importance for the well‐being of society because it is the way to access the largest possible solution space, influence decisions and hold decision takers to account.

The quality of thinking directly shapes the quality of decisions and thus the outcomes that affect people, the biosphere and the planet. In the face of global challenges such as climate change, biodiversity loss, and health crises, cultivating deep, critical, and systems‐oriented thinking is no longer optional; it is essential. Fostering these capacities must begin early in life, within educational systems designed not just to transmit information but to nurture thoughtful, engaged and capable citizens.

## 2 Learning to Think: Development and Maintenance of Thinking Prowess Is Under Threat

Thinking is hard‐wired in the human being (‘Cogito ergo sum’; Descartes; https://plato.stanford.edu/entries/descartes‐epistemology/#CogiErgoSum) but varies enormously quantitatively and qualitatively among individuals, influenced by a range of developmental and contextual parameters that include environment, culture and life experiences. Some of these are:

### 2.1 Questioning and Thinking

According to philosophers, in particular Kant, Leibnitz and Hamilton, everything must have a reason (“Law of Sufficient Reason”; Kant 1787; Baquero 2023; https://plato.stanford.edu/entries/sufficient‐reason/), so understanding is acquired by querying reasons. Our cognitive world might be considered to be a universe of questions. Questioning results in the acquisition of new knowledge; integrating this into the framework of existing knowledge, experience and aims/intentions involves thinking.

Questioning and asking ‘why?’ is a major preoccupation of children between the ages of 2 and 5 (one source suggests they ask some 40,000 questions in this period of their lives: https://amorebeautifulquestion.com/why‐do‐kids‐ask‐so‐many‐questions‐but‐more‐importantly‐why‐do‐they‐stop/), a key element of their developmental programme to comprehend their expanding experience of their world, to build their own framework of truth (Chouinard 2007; Harris 2012; https://www.ascd.org/el/articles/what‐children‐learn‐from‐questioning). After 5, the frequency of questions asked by children reduces as school increasingly demands answers (Susskind 1979; see also video in https://amorebeautifulquestion.com/why‐do‐kids‐ask‐so‐many‐questions‐but‐more‐importantly‐why‐do‐they‐stop/), a trend that continues into adulthood.

This trend is reinforced by active discouragement in many workplaces, where questioning and asking if there is a better way of doing things can result in being labelled as a troublemaker or someone disrespectful of those in authority, rather than as an individual with a positive attitude who can benefit the organisation. As a result, adult behaviour is largely dominated by seeking or producing answers rather than formulating questions.

There are, however, important exceptions to this trend. One is the tradition of questioning in the Jewish faith—‘a faith based on asking questions, sometimes deep and difficult ones that seem to shake the very foundations of faith itself’ (https://www.chabad.org/parshah/article_cdo/aid/3574984/jewish/The‐Necessity‐of‐Asking‐Questions.htm; https://www.jtsa.edu/torah/the‐right‐to‐question/), a public and formal questioning culture that begins early in and continues throughout life. Question formulation also dominates scientific research (Vale 2013), and question‐hypothesis leads to experimental design which is related to the issue of problem solving. This is an important reason to promote science‐technology‐engineering‐mathematics (STEM) interests and careers in school and beyond.

**The Demise of Questioning**

In spite of exceptions, including the existence of internet search engines, questioning, so important for developing thinking in early life, becomes increasingly irrelevant with age for many people, and even a liability in certain contexts. But questioning is not only important for the development and maintenance of thinking abilities. It is vital for our understanding of how things work and what and why changes in the world are taking/have taken/will take place, for democracy and fulfilling our stakeholder responsibilities and holding decision makers to account, and for helping the next generation acquire the ability to navigate the challenges it will face. Society needs a questioning‐thinking reset, with a conscious effort to elevate the role and importance of questioning and debate in daily life. This reset begins in school where discussions and debates that involve active questioning must become normal practice.

### 2.2 Language, Vocabulary and Thinking

Some consider thinking to be a form of ‘inner speech’ or ‘verbal thinking’ a process that ensures the freedom and independence of personal thinking (e.g., Alderson‐Day and Fernyhough 2015; https://www.psychologytoday.com/us/blog/the‐voices‐within/201008/what‐do‐we‐mean‐thinking). For this reason, but also for the broader issues of precision of information processing and granularity/nuances of considerations, language (extent of vocabulary, appreciation, literacy) plays a key role in thinking, cognitive processes and abilities. Since thinking involves using language, the depth of thought and ability to abstract is directly related to the quantity and quality of ‘active’ vocabulary used by the thinker, that is, the number of words and their quality in terms of being able to appreciate and articulate concepts clearly.

Studies of young students have shown that a significant percentage of the population has a very limited and decreasing vocabulary (Milton and Treffers‐Daller 2013; Twenge et al. 2019). This is very visible in texts published on social networks. The downward trend in active vocabulary in society suggests that thinking ability–quality may also be declining.

These issues are compounded in situations where thinking occurs in non‐native languages. Global communication is dominated by a few languages: English, Mandarin, Hindi, Spanish, French, Arabic, Bengali, Portuguese, Russian, Urdu and so forth, obliging many people to communicate, and hence to think, in non‐native languages, in most instances with a restricted vocabulary that restricts their thinking. (That said, there are also significant benefits from thinking in a foreign language: Keysar et al. 2012; Grant et al. 2023; https://www.theguardian.com/science/2023/sep/17/how‐learning‐thinking‐in‐a‐foreign‐language‐improves‐decision‐making).

Given this, it is unsurprising that science—which depends on conceptual precision and a specialised vocabulary—often feels linguistically inaccessible to large segments of society. A narrowing linguistic base amplifies this inaccessibility, hinders public understanding of scientific issues, and limits engagement in informed decision‐making.

In order to protect thinking capacities among nations, there needs to be a concerted pedagogical effort, underpinned by unfettered political support, to counter further losses of vocabulary in adults and enrich it in the young.

### 2.3 Writing and Thinking

Writing compels us to sort and process information, and extract and organise that which is needed for what is to be communicated (https://doi.org/10.1038/s44222‐025‐00323‐4). But the time spent writing, and the quality of writing in general, is in decline (https://literacytrust.org.uk/research‐services/annual‐literacy‐survey/). There is a multitude of reasons for this (https://www.nas.org/academic‐questions/26/3/student_writing_strategies_to_revers_ongoing_decline‐2) but one is undoubtedly the increasing trend to simplify and abbreviate, for example through the pervasive use of emojis in correspondence which, while seemingly cute‐humorous and personal, are in reality impersonal short‐cuts requiring little thought. They constitute a few familiar standardised highly restricted responses to a diverse nuanced spectrum of communications. AI‐formulated standard response prompts are amplifying this disengagement from personal, custom formulations that require thinking; they are substitutes for thinking. As a consequence, writing‐promoted thinking is declining.

Not only this: handwriting, as opposed to keyboard writing, is correlated with greater brain connectivity which in turn is associated with improved memory and learning (Van der Weel and Van der Meer 2024; https://www.scientificamerican.com/article/why‐writing‐by‐hand‐is‐better‐for‐memory‐and‐learning/). But handwriting is a dying art: in many cultures, young people are losing the ability to write by hand (https://www.theguardian.com/news/2025/jan/21/signature‐moves‐are‐we‐losing‐the‐ability‐to‐write‐by‐hand), and, simultaneously the neurophysiological and developmental benefits it brings.

### 2.4 Reading and Thinking

‘Reading develops the imagination. Reading helps you write and speak correctly. Reading takes you places you have never visited. Reading takes you to a time you have never experienced. Reading introduces you to people you have not met. Reading introduces you to new ideas’ (https://literacyblooms.com/reading‐is‐thinking/). Reading delivers knowledge and knowledge empowers. Exercises in the banning and burning of books (https://www.freedomtoread.ca/resources/bannings‐and‐burnings‐in‐history/; https://talkabout.iclrs.org/2019/12/10/1045/; https://www.psychiatrictimes.com/view/the‐psychology‐of‐book‐banning‐and‐burning‐how‐the‐concept‐of‐moral‐panic‐can‐inform‐our‐understanding; https://pen.org/report/banned‐in‐the‐usa‐rising‐bans‐threaten‐1a/), often driven by ‘moral panic’ (Cohen 2011), orchestrated mostly by autocratic and controlling actors in self‐interest, are not only symbolic acts of repression of particular ideas and philosophies; they are explicit acts of censorship, of repression of independent thinking and debate.

Being transported by reading into new situations, other worlds and different historical periods, or being confronted with new ideas, fuels the imagination and drives creative thinking. But, despite the success of e‐books and the convenience of reading on mobile devices, as well as highly visible author prizes (e.g., Nobel Prize; Booker Prize; Pulitzer Prize) and literature fairs (e.g., Buchmesse; Feria Internacional del Libro; La fiera del libro per ragazzi), reading is declining, and reading‐promoted thinking is also declining (https://www.nytimes.com/2025/04/10/opinion/education‐smart‐thinking‐reading‐tariffs.html).

An important factor driving this decline is the trend to visualise information transfer through short, mostly simple videos propagated via social media, and the intense competition for attention inherent in social media (reflected by incessant prompts), which is accompanied by reduced attention spans, all of which reduce demand for the written word and its stimulatory influence on thinking.

Schools (and parents, and society as a whole) urgently need to develop strategies, policies and actions to encourage reading in children (and adults) and promote discussion about what is learned, questioning and debate, and writing.

## 3 Thinking Shapes Identity, Society and the Future

Thinking is significantly influenced/biased by upbringing and life experiences, beliefs, memory, social‐cultural inheritance (e.g., Moore and Brown 2022), the impact of ‘influencers’ and ‘persuaders’ of various types, including but not limited to family and friends, information dissemination operations, fashion and artificial intelligence‐generated messaging. Much of such influence is based upon preformed assumptions that may or may not be valid.

These different variables, and others, and how we learn to think, influence thinking in diverse and complex ways, creating a huge range of individual information handling processes. These in turn determine to a considerable extent what personalities individuals have, to which groupings they belong, which policies and practices they support, and which decisions they make.

**Thinking Shapes Individual Identity, Society and the Future**

The way each of us thinks determines, to a considerable extent, who we are, who we will become, what input we make into matters that involve us, and hence what impact we will have on ourselves and our wellbeing, and that of our family, friends, society and the world. The current state of geopolitics, including conflicts, trade, societal wellbeing, societal inequalities, sustainable development and so forth, is a direct consequence of the way current and past world leaders think/thought. As was succinctly expressed by Jacobs (2016): ‘The crises confronting civilization today are rooted in the way we use our minds—in the way we think’.

## 4 Uncritical Thinking and Information Credibility

Uncritical thinking is the act of accepting information, for example, from family, friends, governments, news outlets, ‘influencers’ (particularly those followed on social media) and, increasingly, from AI services, at face value without significant questioning or verification. Credibility is not a priority or, if it is, is taken for granted because of its real or assumed acceptance by others (Fábián 2015). The personal search for truth is abandoned, delegated to others. Much uncritically accepted information is simple and easy to understand, and hence attractive, but clarity does not guarantee truth. In contrast to the thesis of Descartes (https://www.britannica.com/biography/Rene‐Descartes/Meditations), we must accept that truth is not always ‘clear and distinct’ but frequently complex, contradictory and incomplete, and thus difficult to understand.

Influencers play an increasingly important role in steering thinking, opinion building and development of attitudes in the population. While some influencers promote beneficial opinions, many do not. The problem escalates as uncritical confidence in what opinion leaders say is a contagious process. Moreover, to be fully accepted by friends and colleagues, and make progress in society, there is often an underlying obligation to think as they think. The enormous and catastrophic impact this social contagion has had on wars throughout human history is well documented.

‘Seeing is believing’ is often assumed to be true, but this has never been an absolute criterion: the magician's trade is one of creating illusions, of visible events designed to convince the viewer that something is true or happening that patently is not—‘smoke and mirrors’ (Macknik et al. 2008). More recently, digital tools to create images and cartoons, coupled with the increasing tendency to prefer images over written text to convey information and ideas, have opened powerful avenues of persuasion for uncritical thinkers. Artificial intelligence is an amplifying factor in this (Messeri and Crockett 2024).

**FIGURE 1:**
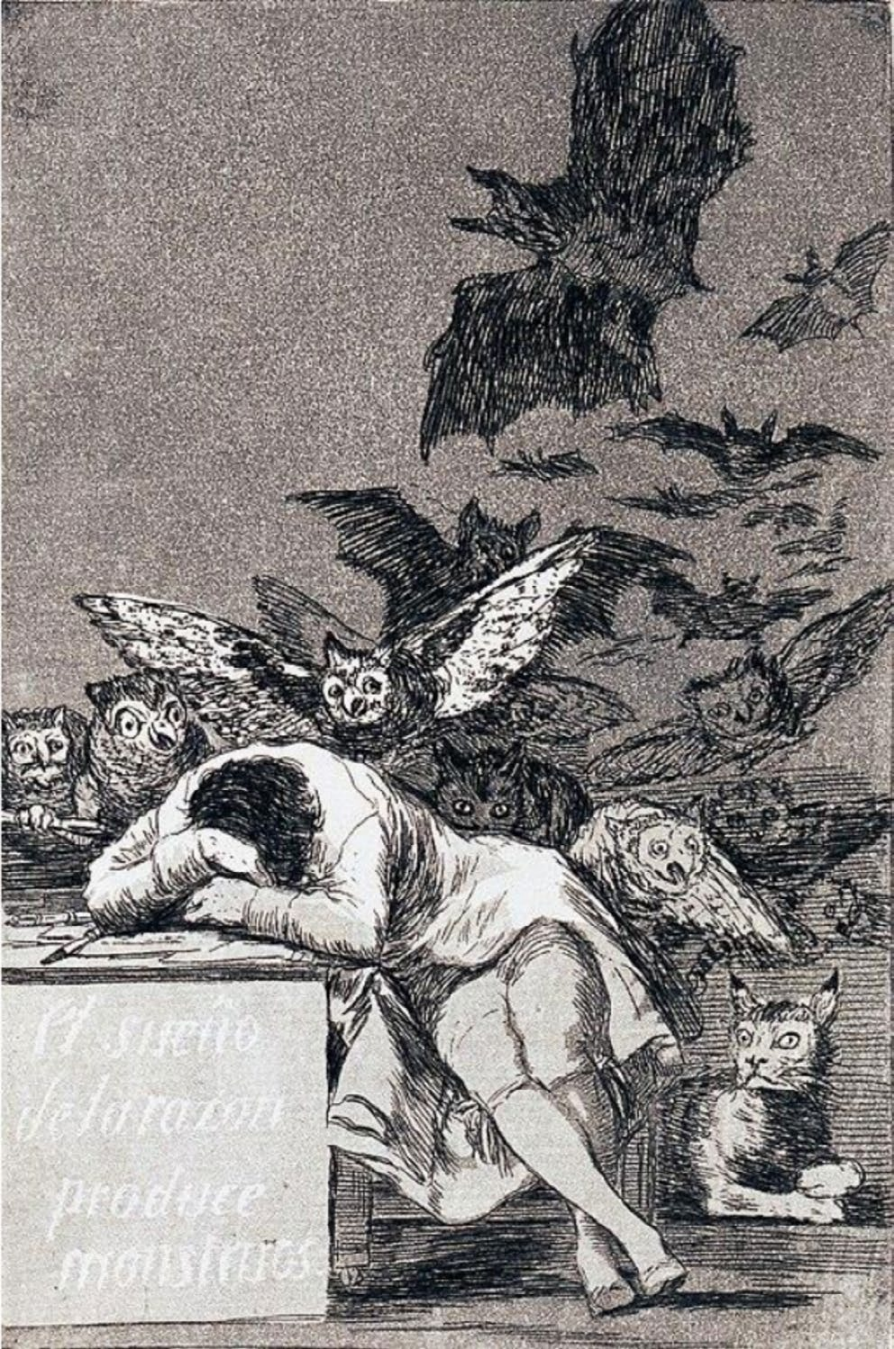
Francisco de Goya y Lucientes (1746–1828)—The Dream of Reason Brings forth Monsters (1799). Museum of Fine Arts, Budapest. *Source:* Jacek Halicki. Francisco Goya, Public domain, via Wikimedia Commons.

It is essential to recognise that the uncritical acceptance of seemingly fair information can create ‘false foundations’ for integrating novel knowledge. In the words of an aquatint of the Spanish painter Francisco de Goya (1799): ‘The dream of reason produces monsters’ (Figure 1).

To address these challenges, we must begin by rebuilding the cognitive foundations of society, starting with education. If we are to cultivate citizens who are able to resist misinformation, engage with complex scientific and societal problems, and contribute thoughtfully to a sustainable future, we must inspire already in young children the satisfaction and value of their personal search for truth and provide them with the tools of critical thinking.

## 5 Independent Thinking Versus ‘Groupthink’, ‘Echo Chambers’ and Indoctrination

Independent thinking and ‘Groupthink’ (Janis 1991) represent opposite ends of a spectrum of cognitive engagement. Independent thinking implies the capacity to evolve different perceptions from those held by the majority, a willingness to question the opinions, advice and assumptions of others, and a capacity to form judgements based on personal experiences and acquired information. Independent thinking is essential to human creativity, progress, and ethical responsibility. In contrast, groupthink constrains and channels thinking, and thereby suppresses independent thinking.

### 5.1 Groupthink and Its Echo Chambers

Groupthink occurs when a desire for conformity overrides individual reasoning, leading to echo chamber groups—groups that engage in discussions which reinforce existing beliefs and disfavour contrary opinions (confirmation bias) leading to a limited perspective and increased polarisation (https://killacodes.com/echo‐chambers‐and‐groupthink‐how‐the‐internet‐shapes‐our‐beliefs/), premature consensus, flawed decisions, and suppression of dissent. Groupthink arises for example when ‘group members value harmony and coherence above critical thought’, often fueled by social pressure or dominant narratives (https://www.psychologytoday.com/us/basics/groupthink; see also Guilbeault et al. 2021). ‘Indoctrination, or forced Groupthink’ is the process of giving repeated instructions or information to a person or a population relating to an ideology with the express intention of avoiding critical analysis. It often implies forms of brainwashing or forced forms of socialisation.

External influences on thinking are often shades of propaganda that seek to undermine independent thought and create groupthink. How serious this can be is exemplified by the promulgation of the discredited assertion that the measles vaccine causes autism, and its exploitation to promote vaccine hesitancy (https://time.com/5175704/andrew‐wakefield‐vaccine‐autism/; https://www.unicef.org/montenegro/en/stories/mmr‐vaccine‐does‐not‐cause‐autism; https://www.dshs.texas.gov/news‐alerts/measles‐outbreak‐2025). Although vaccine hesitancy has a range of causes (e.g., see Heidi et al. 2022; Larson 2022; Jäckle and Timmis 2024), groupthink moulded by anti‐vaccination narratives is important. As a consequence, vaccinations protecting against common and serious infections are in decline and new outbreaks, as exemplified by measles, are on the rise (https://www.cdc.gov/measles/data‐research/index.html). The World Health Organisation has listed vaccine hesitancy as one of the ten threats to global health (https://www.who.int/news‐room/spotlight/ten‐threats‐to‐global‐health‐in‐2019).

The consequences of vaccine hesitancy are readily quantified in terms of morbidity, mortality and the human physical and mental suffering, statistics that need to be considered in context (e.g., it is estimated that the measles vaccine prevented 31.7 million deaths over a 20‐year period https://www.who.int/news‐room/spotlight/history‐of‐vaccination/history‐of‐measles‐vaccination; Venkatesen 2022). The value of vaccines in saving lives and preventing human suffering is extensively and rigorously documented (e.g., see https://www.vaccineseurope.eu/life‐course‐immunisation/value‐of‐vaccination/; Delport et al. 2025). One report suggests that the economic benefit of COVID‐19 vaccines amounted to $US 5.2 trillion (Sevilla et al. 2024). As the World Health Organisation has pointed out: ‘immunization is the single greatest contribution of any health intervention to ensuring babies not only see their first birthdays but continue leading healthy lives into adulthood’ (https://www.who.int/news/item/24‐04‐2024‐global‐immunization‐efforts‐have‐saved‐at‐least‐154‐million‐lives‐over‐the‐past‐50‐years). This attests to the vital importance of routine immunisation schedules for children whose immune systems are not yet fully developed, who do not yet appreciate and implement hygiene practices that reduce transmission of infectious diseases, and thus are particularly vulnerable.

Collaterally, and of considerable relevance to this discourse, vaccine protection of children from disease improves inter alia cognitive functions and academic attainment, that is, thinking capacity (Nandi et al. 2019; Nandi and Shet 2020).

Another obvious example of disastrous groupthink is the denial of the relationship between greenhouse gas production, global warming and extreme weather events (Booker 2018), events that also cause major human and environmental catastrophes like increases in heat‐related deaths, the incidence and severity of wildfires, major flooding events and landslides, that can also be quantified in similar terms of human suffering and environmental damage. One study estimates that by 2050 global warming will have been responsible for 14.5 million premature deaths and economic losses amounting to $ 12.5 trillion (World Economic Forum 2024). Yet still there is an enormous group of deniers, another that is not denying and indeed able to act, but procrastinating instead of acting, and still another—the stakeholders—who fail to hold decision makers to account, all of which contribute to the failure to act promptly and adequately to reduce greenhouse gas emissions.

These are just two examples of multiple global challenges confronted with ineffective responses because of groupthink, despite the huge amount of evidence supporting causality and that constitutes a strong basis for rational thinking and conclusions by society.

Behind many groupthink‐promoting disastrous decisions are other groupthink beliefs, such as the need to discredit everything that provides economic benefits, while disregarding the key role of the industry and business fabric in human development and progress. While economic benefit can indeed be an improper argument of persuasion in some instances, its blanket rejection as a groupthink mantra contradicts reality. Nevertheless, simple and aggressive beliefs are readily propagated.

### 5.2 Independent Thinking

Independent thinkers do not rely on the thoughts and opinions of others but seek, evolve and express their own interpretations of the world. In contrast to groupthink, independent thinking is key to rational discourse and the basis of a high diversity of views and ideas that are vital for the mental exploration of the widest possible idea/options space, human originality and creativity (Monk et al. 2012). Sharing and critically discussing thoughts is essential to making new discoveries. The great philosophers, writers and poets, artists, musicians, scientists and other original creators, who powered the advancement of humanity were/are almost all independent thinkers.

Independent thinking is vital for personal development, the realisation of individual potential, the process of arriving at good decisions, and hence for societal progress. The promotion of independent thinking is especially important in the education of children (https://www.kidsfirstservices.com/first‐insights/why‐it‐s‐important‐to‐encourage‐independent‐thinking‐in‐kids). Schools should not simply convey facts but cultivate learners who are capable of inquiry, skepticism, and thoughtful engagement with complex issues. They should strive to educate all children with the power of self‐confidence to seek truth, solve problems of all levels of complexity and importance, and leverage the diversity of individuals to find inspired, imaginative and adaptive solutions to unexpected changes, to make appropriate and fair decisions. Seeking truth may be a challenge, but it promotes open‐mindedness and independence.

### 5.3 The Danger of Artificial Intelligence (AI): Suppression of Independent Thinking

There are several forces that suppress independent thinking but AI is perhaps one of the most insidious. Its explosive success as a provider of information and online discussion partners/query handlers/problem solvers (chatbots) is remarkable. Its promise of a comprehensive and rational assessment of current knowledge, which it obviously can deliver in an increasing number of spheres, has created a service of convenience that is powerfully addictive. AI is increasingly replacing, and thereby suppressing independent thinking, and in turn influencing the decision‐making process and determining its outcome.

Moreover, as society and family cohesion inexorably declines, societal‐friend‐familial constellations become more fragmented, and support networks fade, companionship AI—replacement of human contact by chatbots—assumes an increasingly dominant role (https://www.youtube.com/watch?v=otAWu‐bLv0Q). Since the provision of companionship AI will be a commercial operation, and retention of clients will be a priority, it is not unlikely that chats will be orchestrated to learn to deliver what the client wants to hear, and hence act as a source of dubious information that reinforces existing biases, as personal echo chambers of thought and opinion. The power of AI to replace independent thinking is huge. As Rainey and Hochberg (2025) have noted ‘From personalised recommendation systems to large‐scale decision‐making frameworks, AI shapes what humans see, choose, believe, and do’. They predict that AI addiction and control of human behaviour is likely to increase through Darwinian evolution of human:AI interactions, a truly frightening scenario.

## 6 Rational Thinking and Evidence‐Basing

Rational thinking is a process that relies on logic and reason. Unlike intuitive thinking, which is fast, automatic, and emotionally charged, rational thinking is analytical, reflective, and evidence‐based. It prioritises coherence and consistency. It eschews hearsay and guesswork, evaluates information, weighs alternatives, and demands that ideas, opinions, and policies be tested, questioned, and revised in light of new information. Rational thinkers do not accept claims at face value; they assess the credibility of sources, identify biases, and seek evidence before reaching conclusions and making decisions (e.g., Grimm and Richter 2024; Toplak and Stanovich 2024). Credible sources of information, that is, those that are evidence‐based, independent, transparent, having a well‐earned/well‐documented reputation for rigor, and that furnish reliable high‐quality materials, seasoned with doubts and uncertainties, which are often ignored by uncritical thinkers, are actively sought.

In complex, high‐stakes contexts—from public health and economics to climate policy and technological governance—rational thinking is not optional; it is essential. When rational thinking is replaced by impulsive reasoning, misinformation, ideological rigidity, short‐termism or seeking personal benefit, the results are often catastrophic: poor policy decisions, societal division, and missed opportunities for innovation or cooperation. Rational thinking is an evolutionary gift and one of the most powerful human assets. Not to cultivate it and use it to the full is a waste of human potential and its capacity to improve the human and planetary condition—a dereliction of duty.

Unfortunately, while rational thinking is a uniquely human capacity, it is not evenly distributed nor spontaneously developed. Cognitive science research shows that rationality must be explicitly taught and nurtured; it does not automatically emerge from intelligence or general knowledge (Stanovich 2009). This underscores the need to embed rational‐thinking skills into the fabric of education, not as peripheral ‘critical thinking exercises’, but as central learning objectives across disciplines (see also the insightful remarks on the challenges facing science made by Adrian Smith, President of the Royal Society, during a recent session of the House of Lords Science and Technology Committee: https://www.parliamentlive.tv/Event/Index/f574c018‐16eb‐4222‐a9c0‐13a215e4322c?_cldee=MTEbxG9f8Q57YOgn06jejg6K8YkV8JwXfKintWxnO_9WbrDZBZc8eCec9kgtukO1&recipientid=contact‐23bef53e7205eb11a813000d3a7f4a6b‐5768695c4dbe46b9a2c51dd10b838392&utm_source=ClickDimensions&utm_medium=email&utm_campaign=Fellowship&esid=a799a16d‐029a‐f011‐b41b‐6045bdfcc2bb).

One crucial enabler of rational thinking is evidence‐basing—the practice of grounding decisions and beliefs in data, observations and empirical validation. Whether evaluating a health claim, a scientific theory, or a public policy, the capacity to distinguish between anecdote and evidence is fundamental. Yet many educational systems still emphasise rote memorisation—imposed ‘truths’—and uncritical acceptance over inquiry, skepticism, and evaluative reasoning—truth seeking. Microbiology is a fast‐moving discipline with new discoveries being made at a frantic pace, some of which upend existing dogmas. An imposed truth today can thus be an imposed lie tomorrow, which underscores the importance of recognising the uncertainty inherent in contemporary knowledge and the need to question its veracity.

A critical element of rational thinking is epistemological literacy, the ability to understand how scientific knowledge is produced, evaluated and revised (and, in some cases, altogether rejected). Evidence‐based reasoning requires an understanding of what qualifies as knowledge or why certain claims gain acceptance. Developing this capacity would allow learners to see Science not as a static collection of facts but as a dynamic process shaped by experimentation, peer review and theoretical interpretation. Without this foundation, students may treat evidence as definitive, rather than provisional and subject to debate (or, again, rejection). Introducing historical case studies—such as discarding the notion of spontaneous generation, the formulation of germ theory or the shift to the heliocentric model—can help to make visible how scientific consensus forms and changes. Historical examples of this sort serve as entry points for understanding the nature of Science while they could encourage critical reflection and prepare young learners to engage with uncertainty in scientific discourse.

## 7 Systems Thinking

We live in an interconnected world in which biological, social, technological and environmental systems constantly interact. As John Muir famously said: ‘When we try to pick out anything by itself, we find it hitched to everything else in the Universe’ (https://vault.sierraclub.org/john_muir_exhibit/writings/misquotes.aspx). Or, in the words of microbiologist Julian Davies (2009): ‘Everything depends on everything else’. This interdependence lies at the heart of systems thinking—an approach that views problems, processes, and phenomena as components of larger, dynamic and interacting systems (Arnold and Wade 2015).

Unlike reductionist or ‘silo’ thinking, which isolates variables in pursuit of simplicity that can lead to linear solutions, systems thinking emphasises relationships, feedback loops, causal complexity and context. When problems are addressed without regard for their broader systemic context, interventions can backfire or produce unintended consequences—an outcome well captured by the ‘Law of Unintended Consequences’ (Merton 1976; see also https://www.sas.upenn.edu/~haroldfs/540/handouts/french/unintconseq.html). For example, treating only the symptoms of environmental degradation without addressing the underlying drivers—such as industrial policies, agricultural practices, energy demands, or the practice of externalisation of costs (‘costs of production that someone else pays’: Timmis, Karahan, et al. 2025; https://realitysandwich.com/sacred_economics_chapter_10/; https://www.imf.org/en/Publications/fandd/issues/Series/Back‐to‐Basics/Externalities)—may exacerbate the problem or displace it elsewhere. The multiplicity of diverse influences that can affect health is explicitly recognised in the One Health paradigm (https://www.avma.org/sites/default/files/resources/onehealth_final.pdf; https://www.fao.org/3/cc2289en/cc2289en.pdf) and the principle of Health in All Policies, which aims to require all public policy deliberations to consider possible health implications of potential decisions (https://iris.who.int/bitstream/handle/10665/112636/9789241506908_eng.pdf). Indeed, because microbes and their activities pervasively underpin many aspects of life, health, the environment, climate, and so on, and diverse policies can impact microbes and their activities, and those of microbe:higher organism holobiont activities, it has been argued that consideration of potential impacts on microbes becomes a similar requirement for public policy deliberations (Timmis, Baquero, et al. 2025).

An important impediment to understanding a phenomenon is incorrect assessment of its position and level in the system in which it is located. Establishment of causal relationships becomes possible only if the phenomenon is classified correctly in terms of its relationships to other elements of the system and the types of interaction it has with them (e.g., see Kotsyurbenko et al. 2020). If secondary interactions are perceived as the main ones, or the phenomenon is considered within the framework of the wrong system, false conclusions are drawn. Moreover, different systems may interact with one another, affecting the phenomenon under consideration and adding a higher level of complexity. For example, the production of methane by microorganisms in wetlands involves the coordinated interaction of different groups of microorganisms that live in the absence of oxygen. However, in the near‐surface aerated layers, methane oxidation rather than methane production is the dominant activity. If the methane oxidisers of a particular wetland intercept most of the methane coming from below, then it might be incorrectly concluded that the microorganisms of the wetland generate very little methane (Kotsyurbenko et al. 2020).

Effective problem solving requires consideration of the entire system, in order to discern root causes, potential solutions and their potential knock‐on effects in other parts of the system (https://ccmit.mit.edu/problem‐solving/). This applies to all manner of spheres of activity (see, e.g., World Economic Forum 2021). In public health, climate policy, urban planning, and education, understanding how actions in one domain cascade into others is essential for effective problem solving. Institutions such as MIT and the World Economic Forum emphasise systems thinking as a crucial 21st‐century skill—one that enables holistic diagnosis, anticipatory decision making, and sustainable solutions (MIT Centre for Collective Intelligence 2021; World Economic Forum 2021).

Despite its importance, systems thinking is rarely taught explicitly in schools. Students are often encouraged to compartmentalise knowledge into isolated subjects, rather than see interconnections across disciplines. As a result, their ability to navigate complexity, reason about causality, and foresee unintended consequences remains underdeveloped.

Microbiology, by its nature, is inherently systemic: microbial processes connect soil, water, human health, biotechnology and planetary systems. Teaching appropriate topics of societally relevant microbiology, as is promoted by the International Microbiology Literacy Initiative and its teaching resources, provides a concrete and engaging way to introduce systems concepts—such as interdependence, cycles, thresholds and network effects—to learners at an early age. In doing so, it equips students with the tools to understand and act within the complex systems that define our lives and future.

## 8 Lateral Thinking and Thinking Outside the Box

Systems thinking requires consideration of the whole picture, not all of which may be fully comprehended, and hence can be challenging. This is especially true of highly complex systems and multiple interacting systems. However, even when there is good comprehension of the diverse components, rational thinking, solutions that work for related problems, and standard routes to solutions may not furnish an answer. In such circumstances, a solution may be revealed by thinking about the problem imaginatively‐creatively in a different, unorthodox way, approaching it indirectly, from a different angle/perspective, seeing other options by blocking out the obvious ones, considering solutions to entirely different problems that have nonetheless something in common with the one confronted, and so forth. Standard solution routes are often programmed by long‐held assumptions which may occasionally channel thinking into unproductive cul de sacs, so challenging or setting aside such assumptions may open up new solution space to explore. Lateral thinkers are typically highly imaginative, and some only think laterally.

The ability to think laterally/outside of the box is a highly valuable quality that is considered to be responsible for many key discoveries in human development and civilisation, and to be a defining trait of the human species. However, it needs an open, flexible and explorative mind, imagination and independent thinking, and may be associated with a higher level of risk. Although natural lateral thinkers possess these qualities, others can acquire them, for example through nurturing imagination infrastructure and problem solving exercises.

**Imagination Infrastructure**

Critical and systems thinking benefit from what may be termed imagination infrastructure—the cognitive, emotional and cultural scaffolding that supports conceptual flexibility, perspective‐shifting and the capacity to generate alternative possibilities (Robinson, König, et al. 2025). This infrastructure plays a foundational role in preparing learners to engage with complexity, particularly in contexts where existing paradigms may no longer be sufficient. In an educational setting, nurturing imagination infrastructure involves cultivating the conditions in which learners are able to explore scenarios, consider unintended consequences and develop the mental flexibility to move between present challenges and potential futures. It strengthens the learner's capacity to analyse, synthesise and construct—to envision new arrangements, relationships and responses within complex systems. Imagination infrastructure is vital.

## 9 Critical Thinking Is Independent, Rational, Evidence‐Based, Systems Thinking

**Critical Thinking**

The *critical* element of critical thinking implies a healthy, reflective scepticism of available information, opinions and advice of others, and promotes constant questioning of the validity of information and opinions. It seeks evidence from credible sources to support ideas and actively explores alternative explanations to those available or newly‐proposed by others. On one hand, it opens the mind to options—perhaps even more rational options—that may not be evident at first sight. On the other, it seeks to recognise and reject—to close the mind to—cognitive distortions (personal and external biases), propaganda, groupthink, emotional manipulation, and the noise overload of irrelevant or misleading information, that interfere with the deliberative process and sound judgement. It is also a barrier to the flood of information and data that dilutes and obscures the key issues and information relevant to informed decision‐making (information pollution: https://www.undp.org/eurasia/dis/misinformation).

Critical thinking is pivotal to good decision‐making and is essential to valid, transparent, accountable decision‐making and decisions that assume the cloak of legitimacy. However, critical thinking ability is not innate; it must be cultivated through deliberate practice and educational design.

Critical thinking unites independent thought, rationality, evidence evaluation, and systems awareness. At its core, it is a self‐directed, self‐correcting process grounded in reason and open‐mindedness. It requires healthy scepticism—not cynicism—toward information, assumptions, and authority. Critical thinkers actively question claims, seek evidence from credible sources, examine alternative explanations, and monitor for bias—both in others and within themselves (Facione 1990). According to Facione (1990) (citation from Aston 2023), ‘CT [critical thinking] is a liberating force in education and a powerful resource in one's personal and civic life… The ideal critical thinker is habitually inquisitive, well informed, trustful of reason, open‐minded, flexible, fair‐minded in evaluation… [and] honest in facing personal biases’.

Critical thinking is directed toward converting reasonable inferences into increasingly solid knowledge. In such a process, a personal ‘integrative imagination’ of what is known and what is possibly expanding the truth, is an epistemological need in which future decision‐makers should be educated. A corollary of this is that critical thinkers are rarely sure of something, since they are cognizant of the possibility that they may not have enough evidence. Paradoxically, because of the realism of critical thinkers (and scientists)—their lack of self‐certainty—they may be regarded as unreliable by those who are used to/are self‐assured.

### 9.1 Critical Thinking May Counteract Neuropsychiatric Issues in the Young Caused By (Anti‐)Social Media Pressures and Misinformation

Young people are subject to sometimes overwhelming pressures and exposure to social media influence, mis‐ and dis‐information (https://www.unhcr.org/innovation/wp‐content/uploads/2022/02/Factsheet‐4.pdf; https://hbr.org/2020/02/the‐era‐of‐antisocial‐social‐media), and peer pressure, all of which can undermine cognitive autonomy (https://www.humanetech.com/brain‐science), and even contribute to anxiety and depression, and sometimes other, more serious, neuropsychiatric disorders (Kelly et al. 2018; Mento et al. 2021; see also Timmis, Karahan, et al. 2025). While the young may be particularly susceptible to peer pressures and the impact of influencers, critical thinking that encourages healthy skepticism of information and evidence‐basing of conclusions, along with the ability to understand and regulate emotions, may reduce their vulnerability and increase their cognitive resilience—the capacity to resist manipulation, tolerate ambiguity, and make reasoned choices in the face of overwhelming input.

**FIGURE 2:**
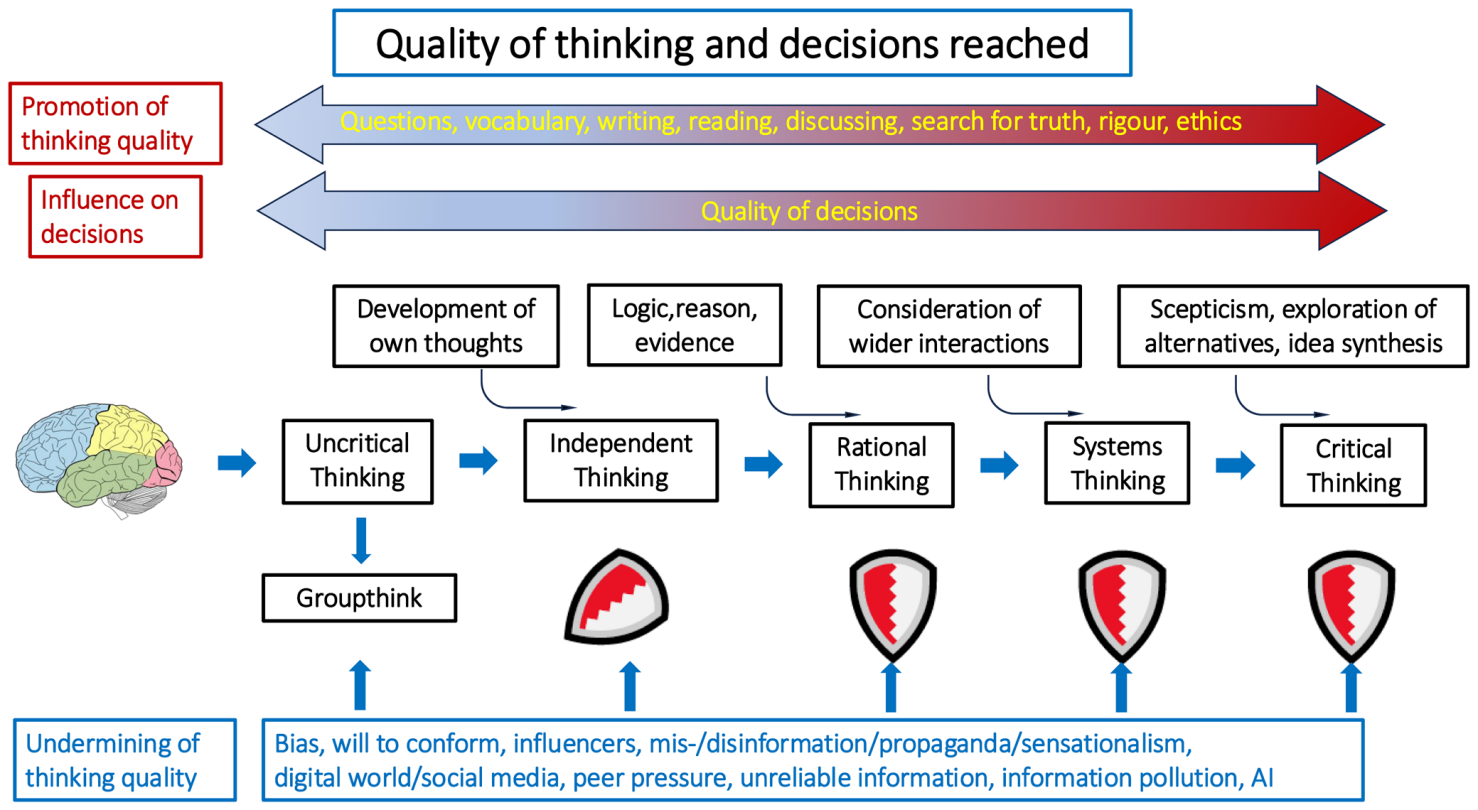
Parameters influencing the quality of thinking and decision making.

In this context, critical thinking becomes not only a cognitive asset but also a protective factor—a shield—for problems of mental health and civic engagement (Figure 2). It prepares individuals to navigate uncertainty, evaluate conflicting claims, and participate meaningfully in democratic societies. Yet traditional curricula often fail to prioritise or integrate critical thinking in a sustained, meaningful way.

## 10 The Vital Importance of Critical Thinking for Effective Decision‐Making at All Levels of Society on All Manner of Issues

Our world—the human, biosphere and planetary ecosystem—is changing at an unprecedented rate and is facing an unprecedented number of crises, some of which are existential, that require effective responses. But while the responses may, in some cases, appear obvious, others are not and, in any case, can involve tortuous political deliberations and decisions that are often biased by short‐term, usually economic, and sometimes vested interests (see, e.g., Monbiot and Hutchinson 2024). Such bias is often compounded by political polarisation, lobbying, prejudice, misinformation and disinformation (Lewandowsky et al. 2017; https://www.unhcr.org/innovation/wp‐content/uploads/2022/02/Factsheet‐4.pdf; https://www.undp.org/eurasia/dis/misinformation), and hearsay and gossip. Factual distortion may be amplified by social media influencing both decision makers and their constituents (Datta et al. 2021; Surjatmodjo et al. 2024). These influences degrade the quality of public discourse and weaken democratic institutions, making it harder for decision makers and citizens alike to act in alignment with evidence, long‐term outcomes, or shared moral imperatives. There is an urgent need for critical thinking—evidence‐based policy‐making and decision‐making at the highest level in order to accelerate the transition of humanity to a healthier, more peaceful and more sustainable developmental trajectory.

It should, however, also be emphasised that not only aspects of the ‘harsh world' of such policy making, market economy, science and technology, but also various aspects of what is called “community goods” such as friendship, belief, trust, collaboration, guidance, constantly need to be subject to critical thinking because they evolve and constitute the basis our “good feeling” in society’.

## 11 Attitudes‐Opinions‐Policy Viewed Through the Lens of Problem Creation and Solving: Tipping the Balance

Almost whenever and wherever people meet socially, conversation drifts inevitably to some current problem or other, its impact and possible solution, and the problem of enacting such a solution: complaining without solving. Many problems experienced by humanity are self‐inflicted by poor quality decisions. Humanity is, for a host of reasons, very prolific at creating problems but less able to solve them. The issues are to improve the quality of decisions, ensure independent assessment of decisions in order to influence their implementation, monitor their success/failure to minimise ongoing, cumulative damage of poor decisions, and repair any damage caused as soon as possible after the event and that caused by legacy decisions. In all cases, critical thinking provides the means of optimising actions.

Humans may be crudely classified into problem creators, problem solvers and those in‐between. The state of the world, nations and individuals reflects to a considerable degree the relative proportions of these three categories. Changing their balance might make a huge difference. Some problem creators may either not know it or, if they do, may not be happy with the situation and would prefer to transit out of this category. Some in‐betweeners would like to be problem solvers if they only knew how. On the other hand, it is unlikely that problem solvers would prefer to be problem creators. Improvement of critical thinking skills in society would provide the cognitive ability for people to become problem solvers and hence drive problem reduction.

A contemporary example of the importance of improvement of critical thinking skills as a means of achieving favourable changes in proportions of societal attitudes is the issue of political populism (e.g., Hunter 2024). Whereas some politicians seek to develop and implement policies that improve the lives of their constituents, others may seek power, personal gain, prestige, or even the worship of acolytes. One means of gaining votes is to embrace populist policies, often by misrepresenting the root causes of problems so as to promote new (own) policies that are different from those in force/promoted by others. For example, the catastrophic humanitarian situation in many parts of the world is driving mass migrations. A key populist mantra is that immigration is bad for constituents because it may place strain on/cause breakdown of infrastructure, result in the importation of different cultures that clash with existing behavioural codes and laws, and increase danger to society. In many cases, the cause of immigration problems is failure to enact policies that provide adequate infrastructure and ensure harmonious integration. Often, inadequate infrastructure was already a problem predating immigration issues (e.g., https://www.cbc.ca/news/canada/british‐columbia/housing‐crisis‐immigration‐1.6878540). By stoking fear and unrest in society, particularly among the poorer, less fortunate, and least well served members of society, and demonising immigrants as competitors for limiting resources, infrastructure and employment, populists create a groupthink that is anti‐humanitarian and gain credence, influence and votes (https://mixedmigration.org/the‐instrumentalisation‐of‐migration‐in‐the‐populist‐era/; Seiger et al. 2025). Critical thinking both fends off misrepresentation and helps people identify true causes of problems, so can drive a reduction in the proportion of those susceptible to populistic propaganda. It may also counter and dis‐incentivise narratives motivated by self‐interest, self‐promotion and desire for unmerited prestige.

Critical and system thinkers are problem solvers. They also learn from their mistakes which, in building problem‐solving skills, is generally considered to be at least as important as, if not more so than, learning from successes. Improving critical thinking skills can change population proportions of groupthinkers to independent thinkers and achieve similar benefits in a range of societal problems resulting from biased and unwarranted attitudes, such as prejudice, stereotyping, discrimination stigmatisation, racism, xenophobia, misogyny, ageism, neurodiversity, stigmatism and sectarianism.

## 12 The Importance of Critical Thinking in Navigating Technological Advances

Humanity is experiencing a period of exceptionally rapid technological advancement that includes the deployment of disruptive and society‐changing technologies such as artificial intelligence, gene editing, synthetic biology, digital surveillance and quantum computing. These advances can create huge benefits for humanity, in terms of economic development and personal wellbeing, and yet may erode critical thinking (George et al. 2024) and create psychological burdens (Liu et al. 2024). However, critical thinking is essential for the ability to analyse complicated discoveries, comprehend how they could affect many spheres including sciences, economics and society, and resolve difficult ethical conundrums. By exercising critical thinking, we may distinguish between trend progression and true breakthroughs, anticipate both the intended and unforeseen effects of technological changes, and efficiently assess the reliability of information in an era of information overload. It also constitutes a defence against lending credibility to ‘novelties’ based on unverified information, thereby contributing to the creation of increasingly false mental structures that lead to deleterious decisions.

In rapidly evolving digital environments, critical thinking fosters intellectual agility and lifelong learning. It allows individuals to update their understanding, learn new tools, and adapt to unfamiliar systems while maintaining a stable core of reflective judgement. As the shelf life of technical skills shortens, the ability to reason, assess and decide wisely becomes a lasting advantage—for individuals, organisations and societies.

In the near future, the ability to collaborate with artificial intelligence systems—becoming what may be termed AI‐augmented strategists—will require technical familiarity and the critical and systems thinking skills necessary to steer innovation toward human and planetary wellbeing. Preparing children for this world must therefore include the cultivation of conceptual flexibility, ethical foresight and the cognitive tools to evaluate and co‐design with emerging technologies.

**The Critical Thinking Compass for Navigation of Complex Issues**

Critical thinking acts as a compass, providing direction while one considers issues such as the moral implications of AI algorithms, privacy issues or the social effects of biotechnology. For ethical and knowledgeable interaction with technology, it is essential to possess the ability for reflection, analysis, and well‐informed decision‐making. A person's ability to prosper in a technology‐driven environment, where being able to navigate complexity and make solid judgements that lead to ethical decision‐making is vital, may be determined by critical thinking abilities.

## 13 Critical Thinking Must Become a Foundational Educational Outcome

From the foregoing, it is evident that a systemic culture of critical thinking—not only among policymakers, but across entire populations is urgently needed. Societies must cultivate citizens who are able to engage with evidence, reason through competing claims, detect misinformation, and hold leaders accountable. Effective, evidence‐based governance requires an informed and critically thinking public. As UNESCO and other global institutions have affirmed, the quality of democratic decision‐making depends directly on the cognitive capacities of its citizenry. Indeed, Rosenberg (2019) has argued that a lack of education frameworks delivering strong cognitive capacity and critical thinking in citizenries is the root of rising right‐wing populism that will eventually displace democracy in favour of autocracy. Development of cognitive skills and capacity for independent and critical‐systems thinking must have the highest priority from the earliest stages of education and become a foundational educational outcome in schools across the world.

## 14 The Case for Teaching Critical Thinking Early in Life

Despite its recognised importance, critical thinking is often taught only as part of university/college‐level education and, in some cases, in higher secondary education. The failure to incorporate critical thinking in education much earlier and more comprehensively is a missed opportunity with wide‐reaching negative consequences. There are several compelling reasons why teaching critical thinking should be taught from early childhood onward:

- Young children are very capable of asking complex, sometimes fundamental questions the answers to which may provoke further questions. How these may be formulated is key to elicitation of satisfactory answers.
- Countering the progressive loss of childhood questioning and desire to understand and influence
- Access and equity: many children do not benefit from a university education or indeed attend higher secondary education, so miss out entirely and become a large segment of adult society lacking notions of critical thinking.
- Early relevance: young children are confronted with many challenges—including peer pressure, fairness, social conflict, digital media content and health‐related information—for which critical thinking would be highly beneficial. Teaching them to reason through options, ask questions, and seek evidence empowers them to handle such challenges with confidence and care (Zacarian and Silverstone 2020).
- Formal education of young children in school, in the sense of providing closed information that is considered the ‘truth’, may repress the natural curiosity of some children, their joy of chance discovery, and acquisition of empirical knowledge
- Cognitive plasticity: young brains are highly malleable. Early exposure to critical thinking can shape lifelong cognitive habits and improve metacognitive regulation—the ability to monitor and adjust one's thinking and actions (Kuhn 1999). In contrast, biases and prejudices, being influenced and persuaded by others, the influence of misinformation, and so on, accumulate with age. Early intervention helps build cognitive immunity to such biases.
- Mental health resilience: acquisition of critical thinking abilities may counter/act as a protective shield against anxiety‐inducing harmful data pollution, social comparison, disinformation and cyber bullying mediated by social media.

To deal optimally and effectively with issues everyone faces from a very early age, it is essential to introduce the teaching of critical thinking as early as possible.

## 15 The Problem of Teaching Critical Thinking in the Young: Conceptual Complexity and Integration in Curricula

Critical thinking is a cognitively complex concept and understanding it is not always easy, even for older children and adults (Gelder 2005; Moore 2011; Davies and Barnett 2015; Aston 2023; https://www.britannica.com/topic/critical‐thinking; https://www.skillsyouneed.com/learn/critical‐thinking.html). Different disciplines may also have different perceptions as to what it is and hence how it should be taught (Jones 2007). In one definition (https://www.criticalthinking.org/pages/defining‐critical‐thinking/766), there is an element of volition ‘based on intellectual commitment’ and personal behaviour ‘a particular way in which information is sought and treated’, ‘mode of cognition’, which suggests that only a subset of a group/class will be able to acquire the capacity for critical thinking. These considerations may imply that teaching critical thinking should only be done once children have reached a developmental stage that allows them to process complex and abstract concepts.

Teaching critical thinking to children thus presents a central challenge in education reform: how do we introduce a cognitively complex skill to learners who may not yet be developmentally ready to fully grasp its abstract dimensions? How do we deal with the conundrum of needing to teach early in life something that can only be properly comprehended later? Another issue is educational context: how to optimally integrate critical thinking into curricula in order to build a robust perception–awareness of the key elements, how they fit together, and how this guides information acquisition and processing, and opinion–judgement–decision formation.

The first issue, the theoretical‐abstract nature of critical thinking, can be circumvented by focusing on practical examples that illustrate the consequences of critical and uncritical thinking in diverse contexts of relevance to children. The second issue is the choice of a disciplinary framework that provides opportunities for discussion of the considerable range of relevant examples needed and that enables coherent integration into school curricula, preferably with added value over and above the intended benefit of incorporating critical thinking.

## 16 Microbiology Is Special—Microbiology Education in Society Is Vital

### 16.1 Microbiology Is Special

Microbiology occupies a singular position among the disciplines: microbes are omnipresent, foundational to planetary health, and deeply intertwined with personal well‐being. Microbiology teaches us the language of life and the laws of the microbial world are highly inspirational in understanding many problems of living beings. Microbes also provide us with a myriad of services and solutions to problems, in addition to annoying us with infections. Microbial activities play a central role in our well‐being, that of all other organisms, and of the planet. Microbes have personal significance: they are our intimate partners that actively share and influence our everyday activities from birth to death (and beyond): they share our meals and go to the toilet with us, share our leisure activities, participate in our romantic activities, and go to bed with us. The impact of microbial activities on the world and its citizens is huge: thinking about us as individuals‐our communities‐the world and how to orchestrate our well‐being through wise decisions, demands knowledge and understanding of microbial processes. As a consequence, societally relevant microbiology is of exceptional importance to society and must become a core subject in education. Importantly, microbiology education is very special—perhaps unique—in readily providing a multitude of examples‐situations that learners can recognise as part of their everyday lives and life experience, and in revealing the pervasive interconnectedness of disparate processes of local, regional and global importance.

### 16.2 The Teaching of Societally Relevant Microbiology Is Special

Fascinating messages that are transmitted include:

- Life began with the microbes who are therefore our ancestors and the organisms that, over millions of years, invented and developed most of our cellular functions.
- Microbes grow on and cover almost all biological surfaces. The communities that cover each organism, including us, constitute ‘microbiomes’. Our microbiome, which co‐evolved with us and our animal ancestors, helps modulate our interactions with the environment, constituting a functional ‘invironment’ that can be considered a human organ, similar to the kidney or the brain.
  Microbiomes interact with their host organisms and provide a wealth of services, such as food digestion, provision of key vitamins and other essential nutrients, protection against invading pathogens, immune training, and so on. When we consume food or take medicines, our gut microbes also participate and influence the outcome of meals and treatments. Our gut microbiota, which secretes molecules that mimic neurotransmitters, is considered to be our second endocrine organ (Clarke et al. 2014), is implicated in our behaviour and mental health, and may contribute to both symptoms and suppression of symptoms of neuropsychiatric disorders.
- Microbes have an amazing range of metabolic activities that enable them to colonise every part of the biosphere (indeed they delimit the biosphere), to metabolise almost all materials that can provide carbon and/or energy, and to produce an unimaginable spectrum of chemicals, some with powerful pharmacological activities, including antibiotics and anticancer agents, organic building materials, materials, enzyme catalysts and degradative activities that have profound consequences for us. They
  ○ Support all forms of life, ecosystems and the biosphere
  ○ Produce much of the oxygen we breathe, play major roles in carbon fluxes that influence global warming and climate change, have created and maintain fertile soils that nourish our food crops, have created carbonaceous geological formations we can see (chalk cliffs) and touch (once processed into travertine tiles)
  ○ Inspire the development of medicines, vaccines, diagnostics, diverse therapies, prophylactics, biocompatible materials for clinical applications, and personal care products
  ○ Produce foods, such as bread and cheese, wine and other fermentation beverages, food supplements, flavourings, and are vital for food security
  ○ Provide agrobiochemicals for crop protection and agents of soil rejuvenation
  ○ Are the main actors in recycling, upcycling and the circular economy, wastewater treatment, water purification, degradation of pollutants (bioremediation), and the recycling of wastes into food in space missions
  ○ Enable the transition from highly energy consuming and highly polluting mining and construction industries to environmentally‐friendly operations (biomining and bioarchitecture)
  ○ Are enablers of paleoanthropology and paleopathology because microbiomes and pathogens can be detected in the remains of our ancient ancestors
  ○ Enable commercial activities and thus provide employment (biotechnology—pharmaceuticals and therapies), chemicals, food and drinks industry, agriculture, environmental protection, biomining, and biotechnology‐related investment/banking services, news outlets (microbial technology is highly innovative with newsworthy applications in many spheres)
  ○ Provide frugal, environmentally friendly solutions to problems that can be afforded by and applied in low resource settings
  ○ Cause disease and deterioration of food and materials, but also serve as natural vaccines, fostering our immunological reactions.

These, and many other aspects of microbiology, such as the diverse roles of microbes in romance and reproduction—activities that significantly preoccupy humans of reproductive age—are easily communicated, readily understood and highly appreciated as being personally and societally relevant, also by the young (Z. C. Karahan, G. Clarke, J. Perlmutter and K. Timmis, in preparation; Robinson, Crino, et al. 2025).

### 16.3 Microbiology Education Is Immersive

Microbiology is practical: many of its messages can be physically demonstrated in class experiments in which learners come into personal contact with study subjects, encouraging observation, questioning, and hypothesis testing. These activities can be further enriched by story‐driven explorations of microbial behaviour, linking scientific processes with narratives of cause and consequence. Class lessons and class experiments may also be complemented by class excursions to experience microbial activities in local settings, such as water treatment plants, bakeries, compositing facilities or local natural ecosystems (McGenity et al. 2020).

## 17 Microbiology Education Is Predestined to Pioneer the Teaching of Critical Thinking in the Young

The immense range of microbial activities that are directly relevant to, and intimately intertwined with, so many aspects of the lives, preoccupations and personal and wider interests of learners, provides an almost unlimited range of ways to personally connect the learner with the subject and to illustrate and promote discussions of the key elements of critical thinking. Few disciplines possess microbiology's capacity to speak simultaneously to personal, local, and global levels of inquiry. It naturally evokes questions that demand critical thinking: How do we balance microbial risk and benefit? What role do microbes play in global sustainability? How do scientific claims about microbiomes relate to commercial interests? These are not just scientific questions; they are civic and ethical ones that stretch and inspire the imagination and stimulate thinking at different levels in multiple spheres.

Because of this unique confluence of practicality, pervasiveness, and pedagogical richness, microbiology is perfectly‐positioned to pioneer a new paradigm of science education: one that does not merely transmit knowledge, but promotes a global view and cultivates thinking citizens—informed, reflective and capable of navigating the complex challenges of the 21st century.

## 18 The International Microbiology Literacy Initiative (IMiLI)

The IMiLI is a global initiative of microbiologists that aims to enable the establishment of a worldwide curriculum in societally relevant topics of microbiology through the creation of a comprehensive collection of teaching resources (Timmis et al. 2019, 2024). IMiLI Regional Centres make available on their websites (https://imili.org; http://www.imili‐eah.com/html/English/EnglishHome.html; https://www.chavarrialab.com/copia‐de‐imili‐cac) the teaching resources in English and regional languages, and interface with educators, learners and education authorities. Completion of current activities to translate the English language teaching resources into Hindi, Chinese, Spanish and Portuguese will make them accessible to more than 50% of the world population. The philosophy of the IMiLI is, like education generally, to provide children with the wherewithal they need to realise fully their potential and become adults able to live productive lives and effectively deal with the challenges they will face by making the best possible decisions. For the reasons given above, and others, microbes and their activities pervasively influence humans, their wellbeing, activities and aspirations; to fulfill its basic goals, education must include societally relevant aspects of microbiology which are pivotal considerations in all manner of decisions.

The core of the teaching resources is the class lessons, designated Topic Frameworks, because they are knowledge frameworks that can be adapted by teachers to different age groups and learning paths (Timmis et al. 2024). Topic Frameworks are learner‐centric, place topics in the wider context such that issues relating to sustainability, stewardship and stakeholder responsibilities are revealed and relevance for decisions becomes apparent. Microbiology‐relevant global and regional challenges are discussed, as are microbial technologies for solving/mitigating them. Topic Frameworks thus not only teach about microbiology but also about the need to improve sustainable development and how this can be achieved, awareness of social inequalities and microbial technologies that can reduce asymmetries, global citizenship, stakeholder responsibilities. These aspects are reinforced by suggestions of class discussions (https://imili.org/topicframeworks).

Topic Frameworks are complemented by the MicroStar Portrait Galleries, portraits of microbial ‘stars’, the actors and their activities discussed in the Topic Frameworks, Class experiments, Class excursions (McGenity et al. 2020), and more. One type of Gallery is the MicroChat Galleries: story‐telling dialogues (sometimes monologues) that explain in story form some important aspect of microbiology. One of these is the Critical Thinking MicroChat Gallery.

## 19 The IMiLI Pedagogical Framework for Teaching Critical Thinking to the Young Through Storytelling

The IMiLI Critical Thinking MicroChat Gallery aims to communicate the practical components of critical thinking, such as the pitfalls and traps that bias or interfere with independent thought (see also Wright 2006, for progress traps), and to provide examples of what critical thinking, and especially what uncritical thinking, are and their consequences, through storytelling (e.g., see https://www.forbes.com/sites/shanesnow/2023/01/16/science‐shows‐humans‐have‐massive‐capacity‐for‐sustained‐attention‐and‐storytelling‐unlocks‐it/). Storytelling—especially when rooted in real‐world problems—offers an age‐appropriate, cognitively engaging pathway to internalise key principles of critical thought, even in the very young (Yaralı and Aytar 2021; Hwang et al. 2023; O'Reilly et al. 2024), without the baggage of the theory. The connection between problem solving (https://ccmit.mit.edu/problem‐solving/) and critical thinking can also be made apparent. For example, stories can illustrate: (1) the danger of following the crowd (groupthink and echo chambers), (2) how assumptions can lead to wrong conclusions, (3) why asking ‘why?’ and ‘what if?’ matters, (4) the consequences of unverified claims.

In parallel, involving students in class discussions of examples of critical thinking reinforces and mentally embeds principles that have been introduced. The same is true of problem‐solving exercises, which require critical analysis and bridge the gap between abstract concepts and practical engagement, laying the groundwork for more formal critical thinking instruction in later years. Both types of exercises engage learners in different ways and challenge them to think more deeply about issues, to reason and develop their capacity to consider alternative views (e.g., Bonwell and Eison 1991; Xu et al. 2023).

By introducing children to microbiological concepts that affect daily life—such as food spoilage, hygiene, disease transmission, and fermentation—the IMiLI creates real‐world contexts for critical reflection. Children explore cause and effect, debunk misconceptions, and question assumptions—all through engaging stories, inquiry‐based learning and problem‐solving tasks.

A scaffolded progression across developmental stages can maximise the impact of early critical thinking education. This progression aligns pedagogical strategies with the learners' cognitive development. Introducing complex reasoning too early, without sufficient cognitive scaffolding, may lead to confusion rather than empowerment. Developmental neuroscience underscores the importance of matching cognitive tasks with children's evolving capacities for abstraction, reflection and synthesis (Gualtieri and Finn 2022). A progressive, age‐appropriate framework can ensure that foundational skills are introduced when they are most likely to be internalised and built upon. For example, children aged 6–9 years can focus on structured observation, pattern recognition, and asking ‘why’ questions to explore cause and effect. Between 10 and 13 years, students can engage in simple modelling, explore multiple explanations and practice identifying assumptions. From age 14 years onward, learners can begin to perform abstract systems analysis, evaluate competing hypotheses and critique knowledge claims. Such a tiered approach supports critical and systems thinking skills, cultivated in a developmentally coherent and cognitively sustainable approach.

## 20 The Practical Elements of Critical Thinking Taught Through MicroChat Stories

What kind of elements of critical thinking can be readily communicated with examples in storytelling, complemented by class discussion and reflection? There are obviously a number, but in Table 1 we list some that are currently being developed that we believe to be of considerable importance, mostly associated with problem solving and proposed change, several of them interconnected. In fact, although presented as separate elements, a number of these are covered by the umbrella issue of reality check/due diligence.

**TABLE 1:** Some elements of critical thinking selected by the International Microbiology Literacy Initiative (IMiLI) to be conveyed through storytelling in its Critical Thinking MicroChat Portrait Gallery.*

Action‐reaction and Gaia
Agendas and hidden agendas
Anthropocentrism
Appearances can be deceiving (due diligence: verify, verify, verify!)
Benchmarking
Best practice
Bias and misinformation
Bottlenecks/limiting parameters
Change: always ask why (if it ain't broke, don't fix it!)
ChatGPT et al.
Consideration of other points of view
Context and its importance
Correlation and causality
Cost:benefit analyses: is it worth it?
Cost externalisation: a reality check of true costs and who pays
Experience in something is not the same as being good at it (talent‐ability‐success)
Influencing, influencers and hype
Interconnectivity/interdependencies: silo and systems thinking
Leading by slogan/motto/cliché/platitude: the persuasive power of words
Operating space and activity constraints
Oversight of decision implementation
Others' shoes (imagine being in them)
Paper trail (who said/did what, when, how, in which context?)
Peer pressure: good and bad
Plausibility and reasonable doubt (does it seem too good to be true?)
Portrayal of issues representing shades of grey as black and white
Predictions, action‐reaction (the Law of Unintended Consequences)
Preparedness vs. self‐fulfilling prophesies
Proportionality
Quality control is essential to the success of decision implementation
Self‐certainty is not the same as being right
Situations and people can change
Stakeholder involvement
Success: has it been defined and can it be measured?
Symptoms versus causes
Talking the talk vesus walking the walk
Transparency, accountability and legitimacy
Trust and healthy scepticism: a prickly issue
Uncoupling from natural checks and balances
Vested interests
Want is not the same as need
Who benefits?

* http://www.imili‐eah.com/html/English/topicFramework.html?resourceId=251&parentId=184&firstTitle=The%20MicroStar%20Portrait%20Galleries&firstTitleEn=The%20MicroStar%20Portrait%20Galleries&secondTitle=The%20Critical%20Thinking%20MicroChats%20Gallery&secondTitle=&thirdTitle=, and below.

To illustrate a few of these examples:

### 20.1 Bottlenecks/Rate‐Limiting Parameters

Sometimes a change or solution to a problem is proposed that may, at first sight, appear rational or attractive (such as increasing consumption of energy drinks to increase athletic performance) when in fact they do not address the key issue (the need for more training to increase muscle mass, strength and cardiovascular parameters). There are many examples of bottlenecks in microbiology, such as eutrophication: the multiplication of microalgae in marine and freshwater systems may be limited by nitrogen—fertilisation of crops leads to leaching of nitrogen into water bodies and removal of the rate limiting parameter—microalgae can then multiply—this leads to a microalgal ‘bloom’, eutrophication and the development of oxygen minimum zones (http://www.imili‐eah.com/prod‐api/profile/upload/2024/11/08/Bottlenecks%20and%20rate%20limiting%20parameters.pdf).

#### 20.1.1 Proposed Class Discussion Topics

Think about and discuss examples of different situations in which rate‐limiting parameters are obviously important, identify them and discuss how to reduce/circumvent/remove them.

Some examples:

- What is the rate‐limiting parameter hindering your favourite football/baseball/hockey team from reaching the top of its league?
- What is the rate‐limiting parameter hindering you personally from doing better at mathematics/geography/and so forth?
- We all need to help with household chores, right? What is the rate‐limiting parameter hindering your ability to contribute more?

### 20.2 Change: Always Ask Why (Is It Really Needed?)

We all know examples of a new boss who starts to initiate changes that seem entirely superfluous (perhaps even harmful), changes seemingly for their own sake/putting his/her stamp on the management ecosystem map. A more positive example from microbiology might be that someone creates an improved vaccine that is less expensive and induces a stronger immune response, and proposes that it should replace an existing vaccine. But the existing vaccine is effective, has good acceptability, and its safety has been documented over many years. SO: while continuous improvement is needed in all manner of things, and a new generation of vaccines may be required in future that will benefit from the progress inherent in the new vaccine under discussion, its perceived benefits may not be a compelling reason to switch.

Another relevant example relates to the discovery of potent and expensive antibiotics. Their use cannot be justified in most infections that can be successfully treated with old, cheap and safe drugs, but a small number of patients might need them to survive. Can we justify the search and development of these super‐antibiotics? Of course, this engages ethical thinking: what is the value of a human life? Do elderly, or severely ill people have less value than the young? This example introduces children to the importance of taking human rights into consideration when making decisions.

### 20.3 Consideration of Alternative Points of View

We always need to consider the possibility that our own perception may not be correct (the healthy scepticism of critical thinking also applies to ourselves!). How to assess this? One good way is to consider alternative views as potentially valid and thus worthy of consideration. One MicroChat illustrating this principle is a monologue by Omi (Omicron variant of SARS‐CoV‐2) explaining why it had unleashed a pandemic, emphasising that we humans carry much of the responsibility, so should not demonise zoonotic viruses, and that infections are just normal ecological interactions that underpin healthy biosphere functions (http://www.imili‐eah.com/prod‐api/profile/upload/2025/05/08/We%20must%20consider%20other%20points%20of%20view%20Omi.pdf).

#### 20.3.1 Proposed Class Discussion Topics

- Do you think that demonisation of Omi and other pathogens causing infections is reasonable? Why or why not?
- Has learning Omi's point of view changed your opinion of pathogens? Why or why not?
- Think of examples of where learning another point of view has changed your opinion about something and explain why.
- Think of examples where putting forward your own opinion to someone, which is different to theirs, might help find a better solution. Explain why this might be the case.
- Do you think that most decisions that affect other people should only be made after different viewpoints have been discussed and considered?

### 20.4 Evaluating Predictions and Misleading Trends

As has been famously stated in various forms: ‘it is difficult to predict, especially the future’. One of the difficulties in predicting is the need to rely on trends, trends that may not continue. As an example, according to the WHO (https://cdn.who.int/media/docs/default‐source/child‐growth/growth‐reference‐5‐19‐years/height‐for‐age‐(5‐19‐years)/hfa‐boys‐5‐19years‐per.pdf?sfvrsn=56a734fa_4), a typical 11‐year‐old boy has a height of 143 cm, which increases to 149 cm by the age of 12, so is growing at a rate of about 6 cm per year. If we predict on the basis of that trend, the boy will have a height of 155 cm at the age of 13, 167 cm at 15, 179 cm at 17, and 191 cm at the age of 19. In fact, the actual numbers are 156, 169, 175 and 176.5 cm, so higher than predicted at 15 and much lower than predicted at 19. The reason, as we all know, is that we go through a growth spurt in mid‐teens and slowdown in late teens, after which we stop growing altogether. So, predicting height increases of children on the basis of trends observed over a limited period of time does not work because our bodies have their own developmental program that ignores mathematical trends.

In weather prediction, trends predicting temperature, wind, or rain for tomorrow are frequently accurate, but less so if we consider weeks or months. Weather is the result of a complex and stochastic association of factors. The same occurs for predicting the emergence of epidemic events.

On the other hand, there are important trends that have excellent predictive value, so we must distinguish those that do from those that don't. In microbiology, dose:response curves can have excellent predictive value that we rely upon almost religiously. In contrast, we may demonstrate that a culture of microorganisms is able to completely degrade a pollutant in a flask under controlled laboratory conditions, but not in the environment, where several variables will influence the process (e.g., adsorption of the pollutant into soil particles, out‐competition of the degrader by other microbes).

### 20.5 Asking ‘Who benefits?’

When people propose a new activity or change in an existing one, they may provide a plausible justification, including possible benefits. However, a proposal may not always be what it seems and may hide benefits to others, including the proposer for personal gain. It is therefore important to seek further information on any other possible beneficiaries (http://www.imili‐eah.com/prod‐api/profile/upload/2024/11/08/Who%20benefits.pdf).

### 20.6 Action‐reaction dynamics

A related key issue is the fact that certain developments do not simply take place in isolation and the world continues its business as normal; very often, an event will provoke a reaction. Action‐reaction is nicely encapsulated in the Gaia hypothesis, which postulates that the Earth's surface is maintained in a habitable state by self‐regulating feedback mechanisms involving organisms tightly coupled to their environment (https://curry.eas.gatech.edu/Courses/6140/ency/Chapter13/Ency_Atmos/Gaia_Hypothesis.pdf). In other words, a change of any significance may provoke a compensatory reaction to restore an optimal equilibrium, a change that often cannot be foreseen.

Feed‐back loops are frequent in biology, and we can conclude that in nature, oscillatory behaviours search for a kind of equilibrium on the basis of coexistence and preservation of diversity. Generally, the relationships between hosts and pathogens tend to equilibrate. ‘Short‐sighted’ microbes are those that, after an apparent benefit of multiplying within a host, kill it and, as a result, may be prevented from reaching other hosts. This impedes their propagation and ultimately results in their extinction.

The emergence and spread of antimicrobial resistance is a bacterial reaction to the use of antibiotics, which also perturbs healthy microbial ecosystems, like human microbiomes.

Another example is provided by tuberculosis caused by the bacterium *Mycobacterium tuberculosis* invading the human lung. The initial innate immune reaction of the host is the formation of granulomas, clusters of immune cells that wall off the bacteria, thereby limiting their spread. Paradoxically, the granuloma may protect the microbe from exposure to other immune defenses. If the infection spreads beyond the granulomas, the host again reacts by recruiting macrophages and releasing cytokines to contain the infection. In response, the bacterium adapts by preventing phagosome‐lysosome fusion in macrophages. The host then mounts an adaptive immune response, activating T cells and producing antibodies. The bacterium counters with surface antigen variation. This action‐reaction interplay results in a complex struggle that determines disease progression, impacting the host's health and immune system equilibrium.

### 20.7 The question of causality

While everything has a cause, a cause generally integrates a myriad of sub‐causes. For instance, we can interpret the decline of *Mycobacterium tuberculosis* infections and deaths as being the result of the discovery of the microbial cause of tuberculosis by Robert Koch in 1880, and the development of anti‐tuberculous drugs around the middle of the last century. However, such a decline started much earlier, around 1850, and should be attributed to improvements in human conditions: reduced malnutrition and overcrowding (McKeown 1979). In other cases, causality is more evident, as exemplified by smallpox vaccination (1786), the drop in infant mortality in the first half of the last century following the application of rehydration procedures, the effect of polio vaccine (1955), or the more recent collapse of *Streptococcus pneumoniae* infections, including antibiotic resistant ones, after the introduction of conjugated vaccine in 2000.

### 20.8 Interconnectedness

Intimately related to action‐reaction is the issue of interconnectivity, the pervasive and inextricable connectedness/network of processes, problems and parameters that affect them, as exemplified by the interconnectivity of poverty, hunger, poor health, poor access to healthcare, poor education, poor access to proper employment, and so forth. Changes—positive or negative—in one component may entrain changes in some or all of the others (see also Sivaramanan and Kotagama 2022), sometimes in unpredictable ways. However, this interconnectedness, and its consequences, are frequently ignored, and problems are often addressed in isolation, without due consideration of either connectivities or collateral effects—the so‐called ‘silo mentality’. When confronted with an issue requiring a change, it is essential to view it in its wider context and explore relevant connectivities, in order to be able to factor potential responses of other elements into the deliberations. Systems consideration and thinking are key to predicting reactions and developing solutions that can yield positive outcomes.

An important example of this is zoonotic infectious diseases: diseases originating in animals that are thought to make up some 60% of all human infectious diseases and 75% of new or emerging infectious diseases (Salyer et al. 2017). Activities that bring humans into greater contact with wild animals, for example by expanding agricultural land into wilderness habitats, ‘wet markets’ selling wild animals, climate and weather pattern changes that alter habitat ranges of animals, or with insect vectors, and so forth, create new conditions for transmission of pathogens from animals to humans: the One Health paradigm. Another example is the use of strong disinfectants in hospitals to kill pathogens that may infect patients. In fact, these select strongly for disinfectant‐resistant microbes and the spread of disinfectant resistance to other hospital microbes. Moreover, and crucially, disinfectant resistance is often genetically linked to antibiotic resistance, so the use of disinfectants in hospitals selects for a hospital‐specific microbial community—microbiome—that is both disinfectant and antibiotic resistant, which is just the contrary to what is intended and needed.

### 20.9 Distortion of deliberations: persuasion, rhetoric, misinformation, influencing, lobbying

Critical thinking deliberately aims to arrive at conclusions that are unbiased, evidence‐based and the best possible result under the prevailing conditions. It is therefore essential to identify any bias, misinformation, undue persuasion/lobbying/rhetoric/narrative hijacking by dominant characters/etc. and eliminate it from the process of arriving at a conclusion. Careful selection of reliable information sources is key. The dangers of following sources propagating false information can be exemplified by the case of the use of oral or parenteral chlorine dioxide or sodium chlorite by patients suspected of, or diagnosed as having, COVID‐19 infections (Chejfec‐Ciociano et al. 2022). Even though the WHO categorically stated there was no evidence to support the use of chlorine to treat COVID‐19, and emphatically warned that the ingestion or inhalation of these products could cause serious adverse effects, the promotion of chlorine use via various channels, mostly social networks, convinced many that it could be an effective treatment.

### 20.10 Transparency and accountability (supported by documentation, where appropriate)

Decisions and solutions to problems can have minor impacts, affecting only one or a few individuals, or major impacts involving large populations and/or the ecosystem in which these individuals are situated. Where a large impact is anticipated, it is essential to create an environment of accountability through transparency of deliberations and the decision process and, crucially, proper documentation and stakeholder consensus. In scientific research, it is standard operating practice to describe in detail the methods employed, to provide access to data and materials used, and to keep a permanent written record of the work. Understanding by other interested parties of how experiments are done and evidence produced, and how results are interpreted and conclusions reached, are de rigueur. Failed experiments and new evidence does not mean that science is wrong, it is how progress is made. Scientific research may thus set the bar for transparency and accountability of decisions and actions.

### 20.11 Specification of measures of success prior to the implementation of decision/solution options

Many decisions‐solutions to problems‐proposals for new actions are simply wrong, either because they are ill‐conceived or simply because the information available at the time was biased or insufficient. In such cases, it is crucial to recognise the mistake as early as possible, so that corrective action can be taken. This requires that unambiguous criteria for success/failure are defined *in advance of commencement of the activity*. In many instances, measures of success of an action/solution are not clearly articulated, which reduces accountability and prejudices objective retrospective assessment of success/utility/effectiveness of an action/policy. *In some cases, failure to specify measures of success, or at least in terms that can be critically evaluated, is deliberate to enable a subsequent claim of success whatever the outcome.* In all cases, it is essential that measurable parameters of success/failure be clearly defined in the proposed plan of action, a schedule specified to assess success at pre‐defined points in time by an independent and competent group of stakeholders, and a plan articulated for corrective action, should success be considered uncertain or unlikely.

Many actions/solutions to problems fail to achieve what they were supposed to achieve, even if they happened to be the best possible at the time and under the prevailing conditions. While this is perfectly normal, it is essential to learn from mistakes in order to avoid repeating them, so an analysis by stakeholders of what led to failure is invaluable.

The storytelling strategy outlined here—grounded in narrative cognition and experiential learning—helps young learners internalise critical thinking principles in a context they can understand and emotionally relate to. It activates sustained attention, empathy, and memory retention, especially when tied to real‐world consequences and follow‐up discussions (Hughes et al. 2022; Eckardt et al. 2024; Maureen et al. 2021). The stories are further enhanced by visual aids, guided discussions, and interactive tasks, allowing learners to explore microbiology content while developing thinking routines, questioning skills, and bias awareness. These examples show that even foundational school science can be a launchpad for higher‐order thinking, provided the pedagogy is aligned with developmental and cognitive science principles.

## 21 Different Considerations of Critical Thinking Intertwine and Combine to Contribute to the Output

In educational and analytical settings, individual aspects of critical thinking—such as questioning assumptions, analysing evidence, or anticipating unintended consequences—are often presented discretely for clarity and focus. However, in real‐world reasoning and decision‐making, these considerations are deeply interconnected and rarely operate in isolation. This interaction is not only inevitable but necessary: the effectiveness of one critical thinking function often depends on the presence or quality of others. For example, a well‐conducted cost–benefit analysis (rational evaluation) may be undermined if the underlying assumptions have not been adequately scrutinised (reality testing), or if stakeholder views have been ignored (ethical and contextual reasoning). Conversely, identifying a flawed assumption may trigger re‐analysis of predicted outcomes, timelines, or measures of success.

This criteria intertwining is illustrated by the example of the consideration of new proposals (Table 2). For example, consideration of plausibility, benchmarking, stakeholder consultation, implementation feasibility, and so on, function best when viewed not as a checklist, but as an interconnected framework because:

- The plausibility of a proposal is shaped by the credibility of sources and the robustness of evidence.
- A cost–benefit analysis draws heavily on benchmarking and measures of success.
- Identifying who benefits might influence judgements about the proposer's motives or track record.
- Weaknesses in monitoring plans may lead to reassessment of timelines or implementation readiness.

**TABLE 2:** A reality check for new proposals.

Plausibility: is the proposal plausible? Has sufficient information been provided to judge it? Are sources credible and unbiased? Has the wider context–interconnectivity been considered?
Compelling case: are the reasons given, and the evidence presented convincing? Are the underlying assumptions explicit, testable and robust?
Alternatives: are there alternative rational proposals that should be considered? Complementarity between new proposals should be examined.
Robust cost:benefit analysis: is it worth it? Risk assessment evaluation.
Benchmarking: is the proposed measure significantly better than the existing best available solution?
Influence: is the new proposal likely to foster similar proposals in other fields, or a chain reaction of decisions? If so, what are the pros and cons of these influences?
Ethics: does a proposal obey the Kantian Categorical Moral Imperative (https://open.library.okstate.edu/introphilosophy/chapter/the‐categorical‐imperative/; https://iep.utm.edu/kantview/), in that it would also have been made if it were to be universally applied, that is, become a universal rule?
Adequate stakeholder involvement: have relevant stakeholders been identified and consulted?
Track record: do you have confidence in the proposer or has a trusted person or expert endorsed the proposal?
Implementation feasibility: is the implementation plan concrete and realistic?
Timetable credibility: is the timetable for implementation and completion plausible?
Measures of success: has ‘success’ of the enterprise been formulated convincingly and measurably, and has success assessment been integrated into the implementation plan?
Monitoring and fallback: is there a progress/success monitoring plan and an escape route if it becomes clear that the plan is unsuitable?

The integrated nature of critical thinking parameters is explicitly demonstrated through progressive storylines and scaffolded tasks. For example, a student might explore a narrative involving the proposal of a new hygiene regulation, evaluating it not only on scientific grounds (evidence) but also in terms of practicality (implementation), equity (stakeholders), and possible resistance (action‐reaction dynamics). Through such integrated learning, students are not just taught what to think critically about—they become equipped to think across domains and make system‐level inferences. When they begin to see these elements as parts of a dynamic cognitive ecosystem, they are better prepared to apply critical thinking to real‐world complexity—whether in evaluating science‐based policies, social dilemmas, or ethical controversies.

## 22 Problem‐Based Learning

Problem‐based learning (PBL) is a pedagogical approach that engages and promotes the development of critical, reflective, and systems‐oriented thinking in learners by challenging them with open‐ended, real‐world issues and difficulties. The students actively participate in researching and comprehending these issues, working together to generate solutions, performing research, and using critical thinking. Individual students and those working in groups may be asked to present more than one possible solution to the proposed problem. This learner‐centred method encourages deep learning, improves students' problem‐solving abilities, and readies them for the complicated and unpredictable situations they could come across in the future. PBL cultivates deeper understanding, long‐term retention, and the ability to transfer knowledge across contexts—all of which are core competencies of critical thinking (Xu et al. 2023; Yu and Zin 2023). PBL enhances not only problem‐solving abilities but also the metacognitive awareness needed to evaluate sources, challenge assumptions, and justify decisions under conditions of uncertainty. Examples include problems based on healthcare scenarios, environmental challenges, business strategies and historical analysis:

- Online games that mimic real‐world problems and provide students with deeper understanding, critical thinking, collaboration, motivation and long‐term learning include, for example, the blood typing game (https://educationalgames.nobelprize.org/educational/medicine/bloodtypinggame/) that effectively describes the concept of blood typing and transfusion without the need for any chemicals. This also sheds light on what happens if a blood transfusion is done with the wrong blood type. Learners must make decisions under time constraints, interpret test results, and avoid life‐threatening errors. The simulation reinforces consequential reasoning—a key critical thinking function—without requiring laboratory resources.
- A community is facing a potential outbreak of an unknown contagious disease: students are required to critically evaluate the available data, consider the implications of different actions, and propose the most effective measures.
- The role of the use of antibiotics in agriculture, farming and aquaculture in the spread of antibiotic resistance to pathogens: students are required to gather evidence to support their arguments, evaluate opposing viewpoints, and develop well‐reasoned arguments.

## 23 Class Discussions, Debates and Reasoning as Critical Thinking Practice

Engaging students in reasoned debate and structured discussion is a powerful pedagogical method for cultivating critical thinking and decision‐making by promoting thorough examination of diverse perspectives. Through dialogic practices, students not only articulate their own arguments but also actively examine and respond to competing viewpoints. Engaging with contrasting viewpoints hones analytical skills, encouraging students to assess evidence, identify biases, and synthesise information. Ultimately, it equips learners to make informed, evidence‐based decisions, fostering a vital skillset for navigating complex issues and contributing effectively to society.

Debate, when facilitated effectively, does more than encourage verbal participation—it nurtures analytical clarity, rhetorical precision, and intellectual empathy. Learners are required to research positions, anticipate counterarguments, and revise their claims based on feedback. This encourages not only cognitive flexibility but also epistemic humility—the recognition that one's own viewpoint may be incomplete or flawed.

Classroom discussions similarly provide space for deliberative reasoning, allowing students to collaboratively test ideas, explore ambiguities, and challenge oversimplifications. Over time, this supports the development of discursive reasoning habits, which extend beyond academic contexts into civic, digital, and professional life. Importantly, it encourages questioning and counters the trend for natural childhood questioning and curiosity to be replaced by an answer‐centric mentality.

A particularly relevant application of debate and discussion lies in the analysis of media narratives, especially those related to science and health. Students may be required to evaluate news or documentaries related to microbiology to identify biases, evaluate the reliability of sources, and differentiate between factual information and misleading interpretations, for example coverage of a vaccine‐related outbreak like measles. Some sources might sensationalise—employing dramatic language without substantial evidence and appealing to emotions—for example by highlighting rare adverse reactions, thereby fostering vaccine hesitancy. Others may downplay risks, potentially overlooking valid concerns. By critically evaluating these perspectives, students discern reliable sources from sensationalist ones and strengthen their ability to differentiate facts from interpretations. The accuracy of claims can be verified by cross‐referencing information from multiple reputable sources. What should be looked for in these resources is well‐substantiated facts, clear references to scientific studies, and a balanced presentation of both potential benefits and risks. Are alternative viewpoints represented? Is the information balanced, or is it skewed toward a specific perspective? Is there a discernible agenda? Does the information provided appear to be cherry‐picking? Is anecdotal evidence used to generalise broader trends? Are fear‐based tactics employed? Recognising these techniques is the key to identifying sensationalism. It should, however, be recognised that finding reliable sources of information is more challenging for non‐English speakers.

## 24 The Wider Ramifications of Teaching Critical Thinking: Advancement of Humanity Through Sustainable Practices and Equitable Sharing of Resources

Economic growth is often portrayed as the solution to a myriad of challenges, including unemployment, cost of living crises, poverty, adequate provision of healthcare services, government borrowing costs, and so forth (Roser 2021; https://drodrik.scholar.harvard.edu/files/dani‐rodrik/files/gcf_rodrik‐working‐paper‐1_‐6‐24‐13.pdf; Fosu 2017). However, much economic growth is fuelled by the input of natural resources that are often limiting, or their access is costly or otherwise constraining (e.g., have knock‐on effects that can increase costs elsewhere, including conflicts, and environmental degradation or pollution). The popular notion that the natural resources Planet Earth can gift humanity are unlimited, as is its ability to tolerate all pollution insults, enabling perpetual economic growth, is an illusion (Meadows et al. 1972; Merz et al. 2023). The less popular notion that perpetual economic growth is neither possible nor desirable (https://www.weforum.org/stories/2022/06/what‐is‐degrowth‐economics‐climate‐change/ https://www.sd‐commission.org.uk/data/files/publications/prosperity_without_growth_report.pdf), and that policies of conservation of natural resources, and strategies to rejuvenate and revitalise the environment instead of exploiting and polluting it, are inevitable for the benefit of future generations, are, however, gaining traction (https://wedocs.unep.org/bitstream/handle/20.500.11822/31813/ERDStrat.pdf?sequence=1&isAllowed=y; https://environment.ec.europa.eu/topics/nature‐and‐biodiversity/nature‐restoration‐regulation_en), though unfortunately not rapidly enough in the body politic. Notwithstanding natural resource limitations, growth in some commercial sectors, especially those that are new and characterised by a high degree of innovation, and corresponding declines in other, older sectors (accompanied by the re‐distribution of resources), are natural outcomes of competitive free markets.

**A Radical Change in Human Behaviour, Driven by Rational, Critical Thinking, Is Vital to Save the Only Known Environment Permitting Life as We Know It**

The originality and creativity underpinning new ideas, that can lead to innovative products and processes which in turn drive economic growth and societal wellbeing, are based upon information processing through independent thinking. Effective information processing necessitates valid information that has been subjected to quality control through filtering by critical thinking. Independent critical thinking is the cornerstone of creativity, originality and innovation in all spheres of human endeavour, including art, literature, science, policy and business. The teaching of critical thinking in school and adult education is of central importance for the healthy development of individuals, the advancement of humanity and society, and sustainability and prevention of degradation and eventual loss of the only known environment permitting life as we know it.

The key to making sure that regional/national economies remain sustainable and do not stagnate as older industries wane is targeting investment to potential growth sectors (e.g., see Timmis et al. 2014). While this can be short term, ultimately it must also become essential long term strategic policy, that is, by providing the necessary education for subsequent generations that underpins its ability to fulfil societal needs (World Economic Forum 2015).

The key to ensuring that under‐resourced economies do not become even less resourced, with global inequalities increasing as a result, is equitable sharing of growth industries and their economic and wellbeing benefits (https://www.unep.org/resources/global‐foresight‐report). Planetary resources belong to all of the biosphere, including humanity, not just the few actors who are nimble and have the necessary technical and financial wherewithal. But the process of equitable sharing involves discussions, choices and decisions on both sides, and fair decisions require a common intellectual language. This in turn requires critical thinking ability by all partners for optimal outcomes. The teaching of critical thinking, which does not involve significant additional costs or investments, is essential in under‐resourced settings; relevant governments must recognise and embrace this and take appropriate education policy decisions. Succeeding to do so will empower future generations and their ability to reduce inequalities.

## 25 Countering Dominant Narratives and Leadership by Slogan/Motto/Cliché/Platitude

Although many important decisions are reached through reasoned discussion in committtee, some committees fail to reach best practice decisions because of a dominant character who determines the narrative. In such cases, the dominant individual assumes/imposes a leadership role and subverts the process of rational decision‐making. The use of slogans, mottos, clichés and platitudes, often emotionally‐weighted (such as ‘It's right for the country’, ‘It's a win‐win (‐win) situation’), are also used widely by leaders to ‘justify’, their decisions and actions, often with little or no evidence. In both cases, critical thinking by those who are ‘led’ can lead to challenges of unreasonable narratives, decisions and policies, and injection of common sense into them.

## 26 Concluding Remarks: The Value of Teaching Critical Thinking in School (and Other Settings)

We have previously argued that *microbes are life* because they are the origin of and sustain all life (Timmis, Baquero, et al. 2025). Here we argue that *thinking is living* because it is the basis of almost all decisions we make, decisions that determine who we are and how we conduct our lives, and our interactions with others and the environment. Critical thinking is widely considered to be essential to objective, evidence‐based deliberation, discourse, opinion‐building, policy development, decision‐making and actioning, and a major bulwark against misinformed and agenda‐led policies and decisions at all levels of society. For humanity to attain higher levels of sustainability, well‐being and inter‐cultural harmony, and deal with the environmental challenges it faces, knowledge and awareness of critical thinking in society must increase as early as possible in the lives of its citizens.

**FIGURE 3:**
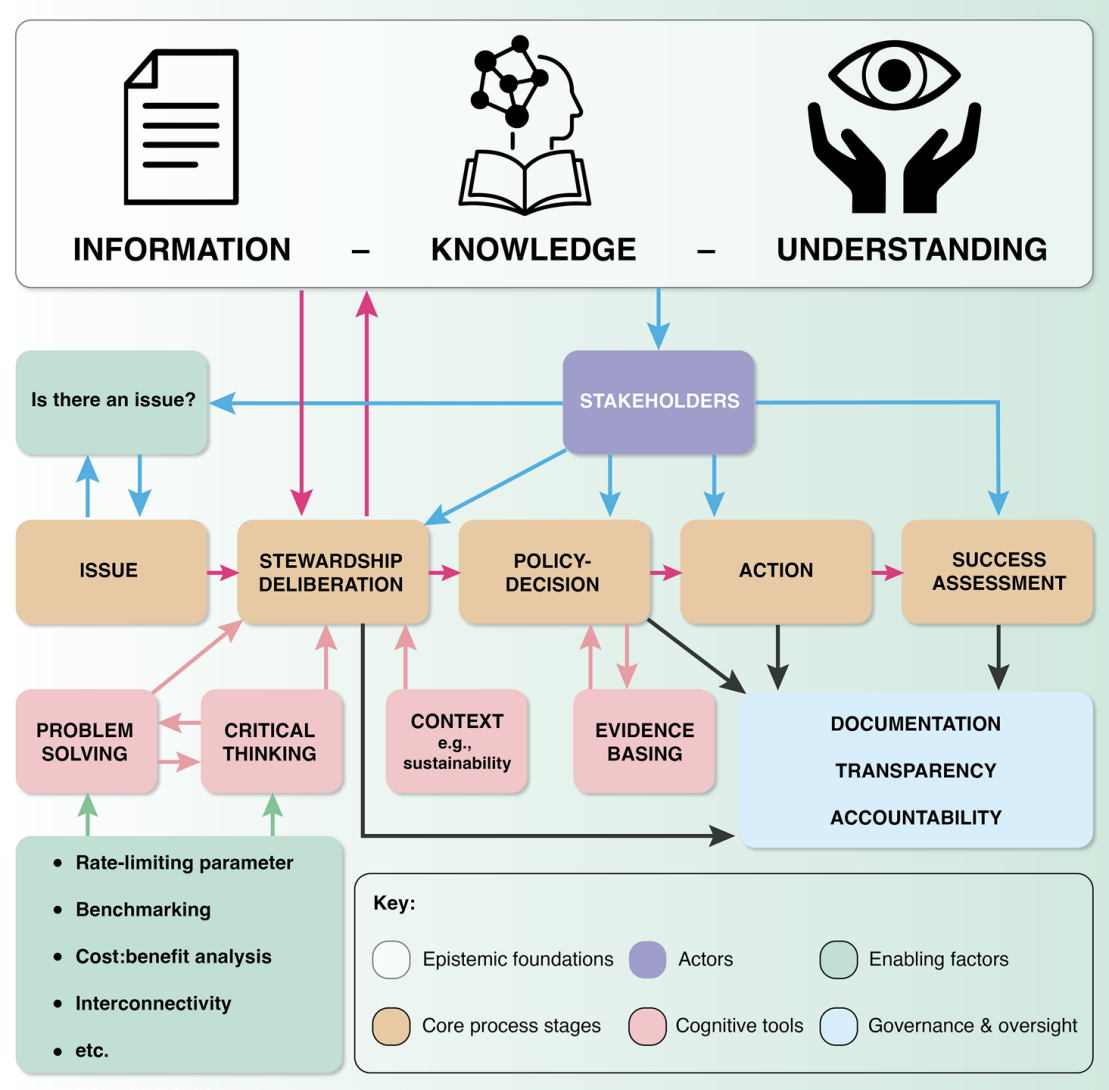
The problem:solution pathway and its key critical thinking inputs that can be learned through storytelling by the young.

While the theory of critical thinking is complex and not readily communicated to young learners, some of its core components—including common pitfalls and traps to be avoided—can be clearly introduced through storytelling based on personally relatable and readily comprehended examples (Figure 3). Contrasting examples of critical thinking considerations with non‐critical thinking and its consequences, and promoting class discussions, debates and problem‐solving exercises on such issues, reinforces central principles. In this regard, microbiology education offers a uniquely fertile context. Its huge range of topics relevant to life, including children's lives, their health and preoccupations, makes microbiology education a perfect framework in which to integrate the teaching of critical thinking. This strategy avoids burdening younger members of society with complex theory, helps them develop key decision‐making and stakeholder involvement skills early in life, and makes the theory of critical thinking that comes later in the curriculum more familiar and readily understandable. Because microbiology is hands‐on, locally observable, and experientially rich, it lends itself to powerful classroom activities that make invisible processes visible and abstract reasoning tangible. Embedding critical thinking elements—such as bias detection, evidence evaluation, stakeholder analysis, unintended consequences, and systems interconnectivity—within microbiology‐based stories, class discussions, and problem‐solving exercises, allows educators to introduce foundational reasoning skills without burdening learners with theoretical abstraction (Figure 3). This strategy not only reinforces central critical thinking principles but also helps children develop essential decision‐making and participatory skills early in life, while making later engagement with formal critical thinking frameworks more intuitive and accessible.

There is currently an epidemic–pandemic of mental health issues in the young. One contributing factor may be the constant and confusing bombardment of mixed messages, disinformation, distorted information, information overload, and algorithm‐driven influencer culture—dynamics that have, to some extent, replaced the transmission of more uniform, culturally constrained information traditionally flowing through parental rearing and education. At a time when youth is increasingly exposed to such cognitive and emotional noise, critical thinking offers more than just academic benefit: it can serve as a cognitive compass, helping learners navigate a world saturated with conflicting messages. Teaching children to question, weigh, and verify information not only fosters cognitive resilience but also supports emotional regulation and reduces susceptibility to manipulation. In this light, education in critical thinking may promote automatic—even unconscious—pathways of evidence‐based filtering of information and thus serve as a partial antidote to the psychological burden of growing up in an environment saturated with unreliable narratives.

As mentioned above, even if critical thinking were to be widely introduced immediately as a core element into school curricula, a vast swath of current society, including many who make decisions and are responsible for policies that affect millions for years/decades, would still lack appreciation for critical thinking and its vital importance. Adults, like teenagers, are overwhelmed with misinformation from social networks, further amplified by TV and radio, making the development of critical thinking skills even more urgent for current generations. Fortunately, there is a resurgence in adult education and adults seeking answers to fundamental questions, so important opportunities also exist for elevating awareness of critical thinking beyond formal education. Of course, the IMiLI stories targeting children may not be appropriate for adults, but the principal elements and examples given are readily repurposed by educators for different audiences.

While the International Microbiology Literacy Initiative is developing stories centred on readily comprehended examples from microbiology, equivalent stories can be readily developed in some other subjects, some of which may illustrate different aspects of critical thinking and thus complement and reinforce those being taught in the context of microbiology (Figure 3). History, literature, environmental studies, and ethics all offer fertile ground for introducing diverse modes of critical reasoning. Cross‐disciplinary integration will amplify learning, reinforce cognitive habits, and normalise critical thinking as a societal value.

Introducing practical elements of critical thinking into school curricula—starting early and across multiple subjects—will bring considerable benefits and significantly raise awareness of its pivotal importance, not only among children but also educators, parents and thence the wider public. And this, in turn, may help broaden the horizons and increase the openness of members of society, counteract the worsening polarities in society, and hence improve both interpersonal relationships and the collective will to build healthier, more cohesive societies.

## Author Contributions

Kenneth Timmis created the first draft; all authors provided input that resulted in the final draft.

## Funding

The authors have nothing to report.

## Conflicts of Interest

The authors declare no conflicts of interest.

## Data Availability

Data sharing not applicable to this article as no datasets were generated or analysed during the current study.
